# Insight on the Interaction between the Camptothecin Derivative and DNA Oligomer Mimicking the Target of Topo I Inhibitors

**DOI:** 10.3390/molecules27206946

**Published:** 2022-10-17

**Authors:** Wojciech Bocian, Beata Naumczuk, Magdalena Urbanowicz, Jerzy Sitkowski, Elżbieta Bednarek, Katarzyna Wiktorska, Anna Pogorzelska, Ewelina Wielgus, Lech Kozerski

**Affiliations:** 1 National Medicines Institute, 00-725 Warsaw, Poland; 2 Institute of Organic Chemistry, Polish Academy of Sciences, 01-224 Warsaw, Poland; 3 Centre of Molecular and Macromolecular Studies, Polish Academy of Science, 90-363 Lodz, Poland

**Keywords:** topoisomerase I inhibitor, DNA, SN38, molecular dynamics, docking analysis, NMR, MALDI-ToF MS

## Abstract

The understanding of the mechanism of Topo I inhibition by organic ligands is a crucial source of information that has led to the design of more effective and safe pharmaceuticals in oncological chemotherapy. The vast number of inhibitors that have been studied in this respect over the last decades have enabled the creation of a concept of an ‘interfacial inhibitor’, thereby describing the machinery of Topo I inhibition. The central module of action of this machinery is the interface of a Topo I/DNA/inhibitor ternary complex. Most of the ‘interfacial inhibitors’ are primarily kinetic inhibitors that form molecular complexes with an “on–off” rate timing; therefore, all of the contacts between the inhibitor and both the enzyme and the DNA are essential to keep the complex stable and reduce the “off rate”. To test this hypothesis, we designed the compound using a C-9-(*N*-(2′-hydroxyethyl)amino)methyl substituent in an SN38 core, with a view that a flexible substituent may bind inside the nick of a model of the DNA and stabilize the complex, leading to a reduction in the “off rate” of a ligand in a potential ternary complex in vivo. Using docking analysis and molecular dynamics, free energy calculations on the level of the *MM-PBSA* and *MM-GBSA* model, here we presented the in silico-calculated structure of a ternary complex involving the studied compound **1**. This confirmed our suggestion that compound **1** is situated in a groove of the nicked DNA model in a few conformations. The number of hydrogen bonds between the components of a ternary complex was established, which strengthens the complex and supports our view. The docking analysis and free energy calculations for the receptor structures which were obtained in the MD simulations of the ternary complex **1**/DNA/Topo I show that the binding constant is stronger than it was for similar complexes with TPT, CPT, and SN38, which are commonly considered as strong Topo I inhibitors. The binary complex structure **1**/DNA was calculated and compared with the experimental results of a complex that was in a solution. The analysis of the cross-peaks in NOESY spectra allowed us to assign the dipolar interactions between the given protons in the calculated structures. A DOSY experiment in the solution confirmed the strong binding of a ligand in a binary complex, having a K_a_ of 746 mM^−1^, which was compared with a K_a_ of 3.78 mM^−1^ for TPT. The MALDI-ToF MS showed the presence of the biohybrid, thus evidencing the occurrence of DNA alkylation by compound **1**. Because of it having a strong molecular complex, alkylation is the most efficient way to reduce the “on–off” timing as it acts as a tool that causes the cog to brake in a working gear, and this is this activity we want to highlight in our contribution. Finally, the Topo I inhibition test showed a lower IC_50_ of the studied compound than it did for CPT and SN38.

## 1. Introduction

Chemotherapy is an effective standard primary medical intervention in combating cancer. Topoisomerase I (Topo I) inhibitors play an important role in this strategy [1,2,3,4]. Topoisomerases are essential enzymes that relieve the torsional stress that is produced in the DNA by cutting the DNA on one strand and thus, allowing the broken strand to rotate around the uncut strand. In human Topo I, this reaction is initiated by Tyr 723, which attacks the phosphate backbone and creates the binary complex with 3′-phosphotyrosine. In the next step, the relaxed DNA is re-ligated by the attack of a free 5′-OH on the phosphotyrosine [4]. As the cleavage step is much slower than the re-ligation step is, the concentration of the nicked DNA is low, but this “on–off” timing is crucial for the inhibition of the Topo I activity. Although several classes of aromatic molecules can intercalate into the nick, thereby forming ternary complex inhibitor/DNA/Topo I, the camptothecin derivatives require special synthetic efforts to be made [5,6]. While the SAR (structure–activity relationship) research reveals the overall activity potential of particular structures on a cell level [7], the molecular insight into the mechanism of action of these potential inhibitors promises progress in us being able to understand the healing process with targeted chemotherapy, which is a key element of personalized medicine. The central point of interest for understanding the process is an interphase of an inhibitor/DNA/Topo I ternary complex [2,4,8]. The stability of this complex is a primary factor, and it is realized through the mutual interaction of complex components. The true inhibitors bind Topo I in a nick, as it has found in a crystal structure of a ternary complex of nicked DNA/Topo I and topotecan (TPT) [2] between 20(S)-OH and Arg533; however, most of the interactions are essentially hydrophobic stacking interactions, which explains the low binding affinity of TPT to the nicked model DNA [9]. Likewise, the majority of the potential inhibitors merely intercalate into a nick of DNA. Pommier et al. developed the concept of ‘interfacial inhibitors’ [10], which is discussed later, in a framework of metadynamics study [11]. These are primarily the kinetic inhibitors that are involved in ternary complexes via a dynamic stacking interaction in a nick with the “on–off” rate timing. Therefore, the primary goal to inhibit Topo I activity is to prolong the “off rate “ timing and prevent the re-ligation of the nicked DNA. It seemed obvious that this can be realized via the substituents’ specific interactions with the DNA in a nick or, most effectively, by covalent binding to the nitrogen bases in the nick faces. Following this idea, the ortho-molecular geometry of the OH and alkylamino substituents in the C-10 and C-9 positions, respectively, of a camptothecin core, may cause the formation of a reactive ‘methide’ intermediate. We proved the covalent binding of such substituted camptothecin derivatives to a model DNA duplex [12], 2′-deoxyguanosine [13], 2′-deoxycytidine [14], and 2′-deoxyadenosine [15]. In order to follow the effects of the site substitution at C-5 and C-9, two series of compounds were studied, and their biological efficiency was established in vitro [16,17]. This reactive intermediate brings about another factor for consideration, which is the lifetime that is required for binding the nitrogen bases in a nick since it easily reacts with water to form a compound with a CH_2_OH substituent at C-9 [18]. In a recent study [19], we have compared the yields of both of the reaction routes, a hydrolysis reaction and alkylation, concerning the secondary and tertiary amine substituents on the C-9 compounds, supposing that the nitrogen geometry can favorably influence the process of alkylation. It became apparent that both of the compounds alkylate the DNA in a nick; fortunately, a derivative with NHCH_3_ at C-9 gave only traces of SN38, which were formed via a retro-Mannich reaction of a parent compound, which is extremely toxic and can cause lethal diarrhea during irinotecan chemotherapy for colorectal cancer. On the contrary, the compound with tertiary nitrogen, *N*-morpholino methyl, gave more of both of the hydrolysis product and SN38.

**Scheme 1:**
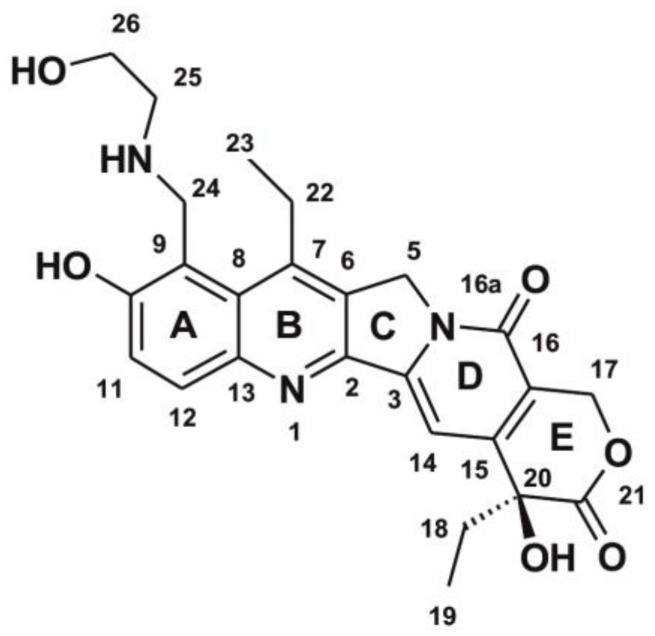
The structure of compound **1**.

**Scheme 2:**
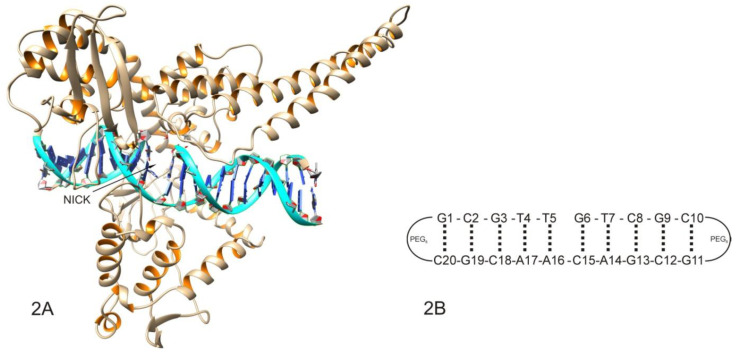
(**2A**) Representation of the biological target of the DNA/Topo I binary complex. (**2B**) The nicked DNA duplex, a model decamer with a PEG(6) tethered at both ends of the duplex. Supplementary Materials, Figure S1. The GC base pair on the right side of the nick (GC side) in nicked DNA portrays the nick environment in wild-type DNA.

Here, we present a study of the interaction of the compound of formula **1** (Scheme 1) with both a biological target, the DNA/Topo I binary complex **2A**, and a model nicked decamer of formula **2B** (Scheme 2) using the molecular modeling and experimental results. It was presupposed that the hydroxyl group on a long alkylamino chain may have better hydrogen bonding abilities and they may strengthen the molecular complex with **2A** and **2B**. The preliminary PFGSE (pulsed-field gradient spin echo) experiment confirmed a strong interaction of compound **1** with **2B** molecules. Therefore, it was reasoned that this compound has two properties which are conducive to prolonging the ‘off’ timing of the molecular complex: the potential alkylation via the C-9 alkylamino substituent and its hydrogen bonding abilities inside the nick.

## 2. Results and Discussion

### 2.1. Molecular Docking Analysis

Computational docking studies were performed to predict the mode of binding of compound **1** to the nicked DNA/Topo I complex (**2A**). The open-source AutoDock software package was used for the research, including AutoDock-GPU, the Cuda-accelerated version of AutoDock 4.2 [20]. The two X-ray structures that are available in RCSB (1K4T and 1T8I) of the ternary TPT/DNA/Topo I and CPT/DNA/Topo I complexes were used as the receptors, after the removal of the ligand components (TPT or CPT). During the first attempt, the standard docking procedure with the rigid receptor and ligands with flexible bonds and the Lamarckian genetic algorithm with an empirical free energy scoring function were used. The results of this docking procedure are shown in Table 1 in the “X-ray single structure” column. In addition to compound **1**, the results of the docking calculations were also presented for several other reference compounds such as camptothecin (CPT), topotecan (TPT), and SN38. The best docking poses for all of the ligands were identical and they were similar to the conformation of the ligands (TPT and CPT) in the X-ray structures. Interestingly, the best binding energies were observed for compound **1** and they gradually degraded in the order: compound **1**, TPT, CPT, and SN38. To improve the accuracy of the docking calculations in the next calculation attempt of the receptor model, the structures that were acquired during 500 ns explicit-solvent molecular dynamic calculations (MD) were used. The application of the molecular dynamics simulations enabled an evaluation of the receptor side-chain and backbone movement within the complex during the docking, in principle, allowing the generation of novel conformations. Two molecular dynamics trajectories were carried out for the 1K4T and 1T8I X-ray-starting structures, and 1000 structures were uniformly recorded during the MD run for each of them. After the removal of the TPT or CPT ligands, the recorded structures were used in the docking calculations. The results are presented in Table 1 in the “1000 MD derived structures” column. The lowest binding energy was observed for compound **1**, which was followed by SN38. These results were surprisingly consistent with the experimental data (Figure 6) on the inhibition of the human Topo I relaxation activity. Again, the best docking poses were analogous to the X-ray structures. However, a careful conformation analysis which was performed by the clustering method suggests that other distinct ligand conformations with respect to the receptor were also present, especially for compound **1**.

**Figure 1:**
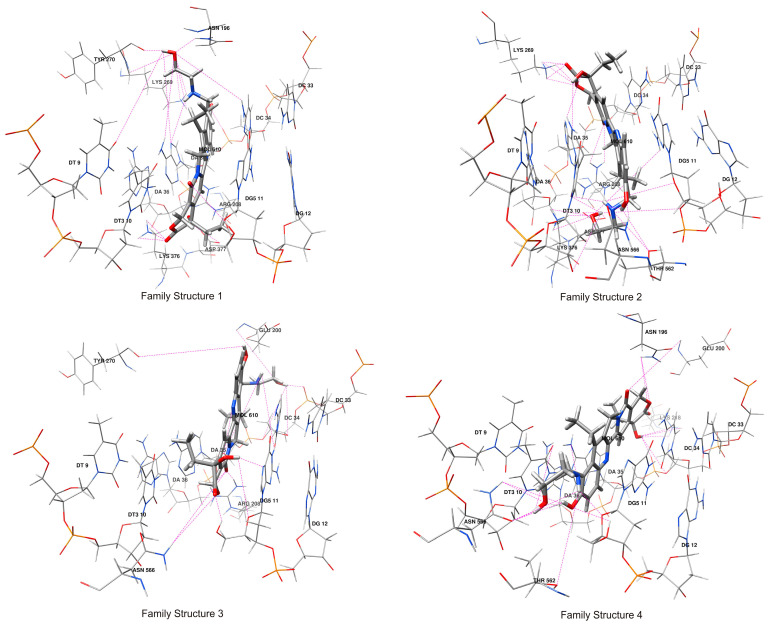
Structures representing the main clusters for each family structure obtained in MD simulations. Only ligand-interacting fragments of the receptor are shown. Dashed purple lines indicate possible hydrogen bonds for a given family of structures.

The above docking calculations clearly artificially favored the formation of a ligand pose that was consistent with the X-ray structures. To prevent this, new molecular dynamics calculations were performed for the compound **1**/DNA/Topo I ternary complex. This time, starting from the X-ray structure of the TPT/DNA/Topo I complex (1K4T), the TPT ligand was replaced with compound **1**. However, the new ligand was placed at all of the four possible distinct positions that were relative to the receptor. For each of the four obtained complexes, two separate molecular dynamics simulation trajectories were produced. This resulted in a total of eight 500 ns trajectories. As previously stated, 1000 structures were uniformly recorded during the MD run for each of them. For the 8000 receptor structures that were thus obtained (after the ligand removal), the docking calculations were performed. Then, the best docked structures were divided by a cluster analysis into four structure families that were analogous to those in the MD calculations. Family structure 1 was consistent with the X-ray structures, family structure 2 represented the structures with a ligand that was rotated 180 degrees about an axis which was perpendicular to the artificial plane of the molecule, family structure 3 represented the structures with a ligand that was rotated 180 degrees about a long axis parallel to the artificial plane of the molecule, and family structure 4 represented the structures with the ligand which was rotated 180 degrees about both of the above axes (Figure 1). For each of the families of the structures, 50% of the structures with the highest energies were rejected, and for the rest, the mean binding energies and the mean values of the inhibition constant were calculated. A similar docking procedure was used for the reference compounds. However, it should be noted that, with these reference compounds, the calculations are somewhat less reliable as only the receptor structures from the MD calculations for compound **1** were used. The results are presented in Table 2.

By far, compound **1** binds the most strongly to the receptor when it is compared to the reference compounds, and the computation unexpectedly favors the family structure 2 complex formation. While for TPT, CPT, and SN38, the family structure 1 is definitely preferred, which is consistent with the current knowledge, including over the X-ray structures [2,21]. However, one has to be aware that the calculated binding energies do not take into account the effect of the entropy. Following the method that was proposed by Ruvinsky, it can be assumed that entropy is somewhat proportional to the size of the structure cluster (CS) [22]. The entropy contribution for TPT, CPT, and SN38 are always the largest for the family structure 1 and this does not significantly a change to our conclusions. For compound **1**, the comparable sizes of the clusters for the families of structures 1 and 2 were observed. It can, therefore, be concluded that, for the compound **1**/DNA/Topo I ternary complex, family structure 2 is preferred; however, it cannot be ruled out that it is in dynamic equilibrium with family structure 1.

### 2.2. MM-PBSA and MM-GBSA Calculations of the Ligand/DNA/Topo I Ternary Complex

With the molecular dynamics simulation at hand, the molecular mechanic’s ligand binding energies which were combined with the Poisson–Boltzmann or generalized Born and surface area continuum solvation methods (MM-PBSA and MM-GBSA) were calculated and successfully used to reproduce and rationalize the experimental findings and to improve the results of the virtual screening and docking. The calculations contain several crude and questionable approximations, for example, there is a lack of conformational entropy and information about the number and free energy of the water molecules in the binding site. However, the biggest problem seems to be the need for a sufficiently accurate sampling of the conformational space, which is usually associated with very long molecular dynamics simulations. To monitor whether the molecular dynamics were simulated to a sufficient extent, our simulations were carried out on two independent trajectories (A and B) for each family structure of the compound **1**/DNA/Topo I ternary complex. For each trajectory, 25,000 samples of structures were used for the calculations (see Table 3). Unfortunately, the calculation results for trajectories A and B were sometimes very different. This is most likely due to the fact that the molecular dynamics simulation that was 500 ns long may still be far too short and/or the systems are not in equilibrium. Nevertheless, one can justify the assumption that the preferred conformations for the compound **1**/DNA/Topo I complex are those for family structure 1 and are less likely for family structure 2. The compound **1**/DNA/Topo I complex seems to be the most strongly bound, despite the fact that comparing the relative binding energies between the complexes is less reliable.

### 2.3. Hydrogen Bonds Analysis of Compound **1**/DNA/Topo I Ternary Complex

Intermolecular hydrogen bonds (HB) play a very important role in ligand–receptor interactions because they usually make a significant energetic contribution to the binding energy [23]. The statistics of these HB bonds are presented in Table 4, and in Figure 1 in a graphic form. Table 4 presents the statistics separately for each family of structures and it does so separately for the structures that were obtained from the docking calculations and the molecular dynamics simulations. The statistics include the average length of a given hydrogen bond and its population, i.e., in what percentage of the structures that a given bond is observed. Once again, it can be observed that family structure 2 is preferred, and family structure 1 is slightly less preferred. Interestingly, approximately half of the hydrogen bonds are formed with the hydroxyalkylamino substituent at the C-9 position of compound **1,** which confirms our hope that its introduction into the molecule will significantly increase the topoisomerase I binding ability.

By analyzing Table 4, it can also be seen that more hydrogen bonds are usually observed for the structures that were obtained by docking. It can be suspected that this is due to the insufficient sampling of the conformational space during the MD simulation or some shortcomings of the docking protocol (for example, no consideration of the explicit solvent). Nevertheless, the convergence of the results for both of the methods is quite good, especially for the preferred family structure 2.

### 2.4. The NMR Results of TOCSY and NOESY Experiments for the Binary Compound **1**/DNA Complex

In order to prove our hypothesis and determine the geometry of the complex, the NOESY and TOCSY experiments were performed. These 2D NMR experiments are commonly used in the proper assignments of the proton DNA signals and also to study the DNA interaction with the ligands. Based on the induced changes in the chemical shifts of the ligand and the DNA signals, and the presence of the intermolecular cross-peaks (the correlation through the space within approximately 5 Å) between the hydrogen atoms in the different molecules, we can obtain the information regarding where and whether or not there is an interaction between them.

**Figure 2:**
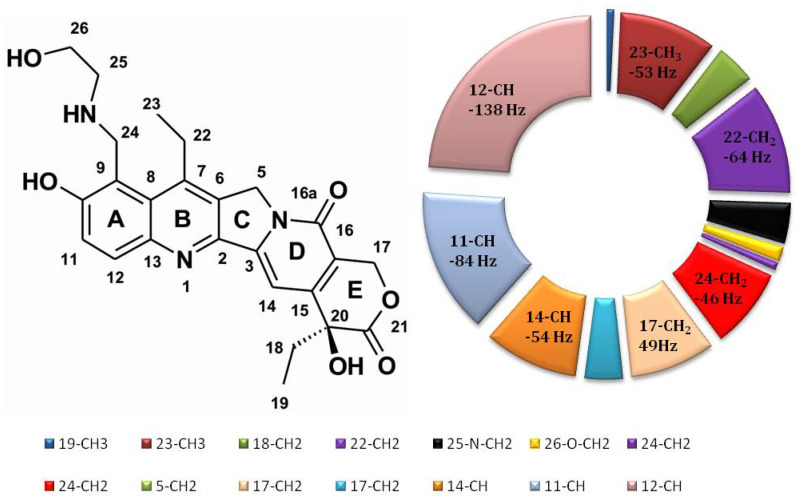
The ^1^H NMR chemical shift changes in compound **1** after addition to DNA (**2B**).

The NOESY and TOCSY spectra (Figures S3 and S4) were used to assign the proton chemical shifts of compound **1** and decamer **2B** in a formed complex (Tables S1 and S3), which included an assignment of all of the found intermolecular cross-peaks. Figure 2 shows the changes in the chemical shifts that were induced in the protons in compound **1** by its interaction with **2B** in a complex, and Table 5 shows (Table S2) the changes that were induced in the chemical shifts of the neat decamer **2B** by its interaction with **1**. As can be seen in Figure 2, the biggest changes in the chemical shifts for compound **1** are observed for the protons of the aromatic rings A and B, e.g., 11-H and 12-H, indicating that the aromatic core is involved in an interaction with the DNA (2B). As the largest chemical shift changes in the **2B** protons are observed on the base pairs nearby the nick and most of the protons undergo low-frequency shifts, the presented data indicate the stacking interaction between the aromatic core of compound **1** and the nitrogen bases flanking the nick faces of the DNA.

**Figure 3:**
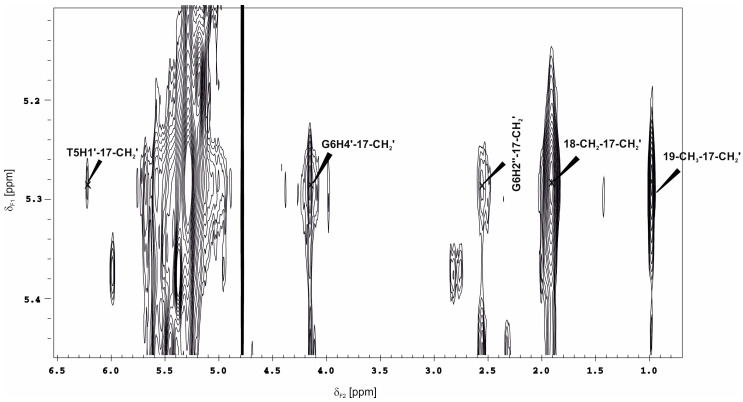
Part of the NOESY spectrum represents the intermolecular cross-peaks between the 17-CH_2_ group in compound **1** and DNA bases in the nick of **2B**.

The strong interaction between the two molecules allows for the observation of the intermolecular cross-peaks that are shown in Figure 3 (Figure S5) and Table 8. The obtained results of the NOESY experiments were also used for their comparison with the results of the MD simulations for the compound **1**/DNA binary complex.

### 2.5. MM-PBSA and MM-GBSA Calculations of the Compound **1**/DNA Binary Complex

For smaller systems, calculating the molecular dynamics of them is easier. Not only does the speed of the calculations themselves increase, but it is also easier to obtain an accurate sampling of the conformational space due to there being a lower number of degrees of freedom. That is why the molecular dynamics simulations were performed, and these were followed by the calculation of the binding free energy (MM-PBSA and MM-GBSA) for the much smaller binary compound **1**/DNA complex. The simulated binary compound **1**/DNA complex was exactly the same complex as that used in the NMR measurements. This allows for a better interpretation of the experimental NMR measurement results. Furthermore, this time, the molecular dynamics simulations were performed for four families of the structures, with there being two trajectories for each. However, the simulations were extended to 1000 ns. The results of these calculations are presented in Table 6. The quality of the calculations was clearly improved; however, there are still significant differences between some of the A and B trajectories. Moreover, the simplification of the complex evidently changed the order between the families of the structures. The lowest free energy of binding value was observed for family structure 3, which was followed by family structure 4.

### 2.6. PM7 Semi-Empirical Calculations of the Truncated Compound **1**/DNA Binary Complex

After truncating the DNA to only two base pairs so that compound **1** was complex with there being one GC and one TA base pair on both sides of the nick, it was possible to perform semi-empirical calculations using the PM7 method. The 5000 samples of the structures for each trajectory were used for the calculations, and the water solvent was approximated with the COSMO model. The results are presented in Table 7. Due to the fact that the PM7 calculations included the geometry minimization step, the results of the calculations do not seem to be burdened with the problem of the insufficient sampling of the conformational space. Unfortunately, the large standard deviation of the results and the very large simplification of the model means that a careful interpretation of the results is required. However, it seems reasonable to confirm the observation that the family of structures 3 is preferred based on these calculations.

### 2.7. Comparison of NOE Effects with MD Simulations for the Compound **1**/DNA Binary Complex

The third column of Table 8 shows the intermolecular NOE effects that were measured in the NOESY NMR experiment. These effects are between compound **1** and the DNA hydrogen atoms, and they are scaled to the cytidine H5/H6 protons’ NOE average effects. The presented percentage values of NOE effects are rather qualitative as they may be disturbed with a large errors due to the spin diffusion and the dynamic exchange effects that occur. However, an attempt was made to correlate them simply with the geometrical data that were obtained during the simulation of the molecular dynamics. The symbol ++++ in Table 8 means that a significant amount (>50%) of the sampled structures met the given distance condition (e.g., <6 Å). This directly means that for a given pair of protons, the NOE effect should be observed, with a value that is proportional to the given distance criterion. This value, by simplifying most often, is proportional to 1/r^6^, where r is the interatomic distance. In general, when a large NOE effect is observed, a large number of + symbols should also be observed for the short distance criteria when the family structure geometry is right. In summary, this analysis shows that family structure 1 correlates best with the NOE data. However, the presence of family structure 3 cannot be rejected as the spin diffusion has the potential to significantly overestimate the number of NOE effects that are observed [24]. This is important because the NOE spectra had to be recorded using relatively long mixing times in order to achieve a sufficient signal-to-noise ratio. It is also possible that both of the families of structures are present in dynamic equilibrium.

**Figure 4:**
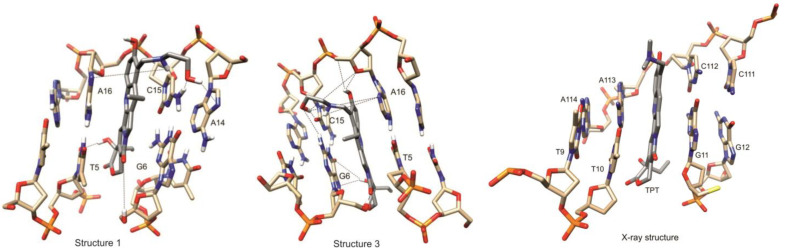
Structures representing the main clusters for family structures 1 and 3. Dotted lines indicate a possible hydrogen bond network stabilizing the structure. For clarity, only two base pairs flanking the nick are shown. Right panel shows the snapshot from the X-ray complex of nicked 20-mer DNA with topotecan [2].

The structures in Figure 4 show the two most abundant representatives of the clusters describing a molecular complex for family structures 1 and 3 in solutions. Both of the structures are stacking inside the nick with a ring of camptothecin facing the unbroken strand of the nicked DNA. In accordance with our expectations, the long aliphatic chain in the C-9 position with NH and OH hydrophilic groups play an important role in stabilizing the molecular complex because both of them act as donors and acceptors of the hydrogen bonding. The aliphatic chain is displaced along the base pairs in a minor groove in structure 1 and along the major groove in structure 3 due to the fact that the CPT core is turned over 180 degrees in both of these situations. Both of the computed structures in Figure 4 are in agreement with the experimental results that are given in Figure 2 and Table 5 and the observed cross-peaks that are given in Table 8.

### 2.8. DOSY Experiments of Molecular Complexes

Diffusion-ordered spectroscopy (DOSY) is an NMR experiment that is widely used for the investigation of the binding affinity of the interacting molecules in the molecular complexes that are in solutions [25,26]. The small ligand **1** will have a much smaller diffusion coefficient upon their binding interaction with the larger DNA molecule **2B** when they are compared with the diffusion coefficients that occur when it is unbound by measuring the diffusion coefficients D_i_ × 10^−10^ (m^2^ s^−1^) of both of the free and complexed compounds. Consequently, we are able to establish the binding constant (see Section 3). Table 9 shows the data that are used to calculate the binding constant of a complex. From these data, it appears that almost all of compound **1** is involved in a complex with DNA **2B**. The DOSY spectra are presented in the Supplementary Materials (Figures S7 and S8). The results that were obtained for the molecular complex (**1** + **2B**) show that the interacting molecules form a very strong complex with the calculated value of K_a_, which is equal to 746 mM^−1^ (Table 9). By comparing these results with the previously determined K_a_ value for the topotecan complex with the same DNA oligomer (**2B**) [9], with a K_a_ of 3.78 mM^−1^, we can assume that compound **1** has a significantly better binding affinity for DNA than topotecan does. It is most likely that this could be explained by the better ability of compound **1** to create hydrogen bonds with DNA. The results of the presented hydrogen bond analysis proved this hypothesis, and they show that the hydroxyalkylamino substituent at C-9 of **1** contributes to approximately half of the hydrogen bonds in the compound **1**/DNA/Topo I ternary complex. Moreover, of the seven presented hydrogen bonds in the complex, five of them are involved in the formation of family structure 1 (Table 4).

### 2.9. MALDI Experiment 

**Figure 5:**
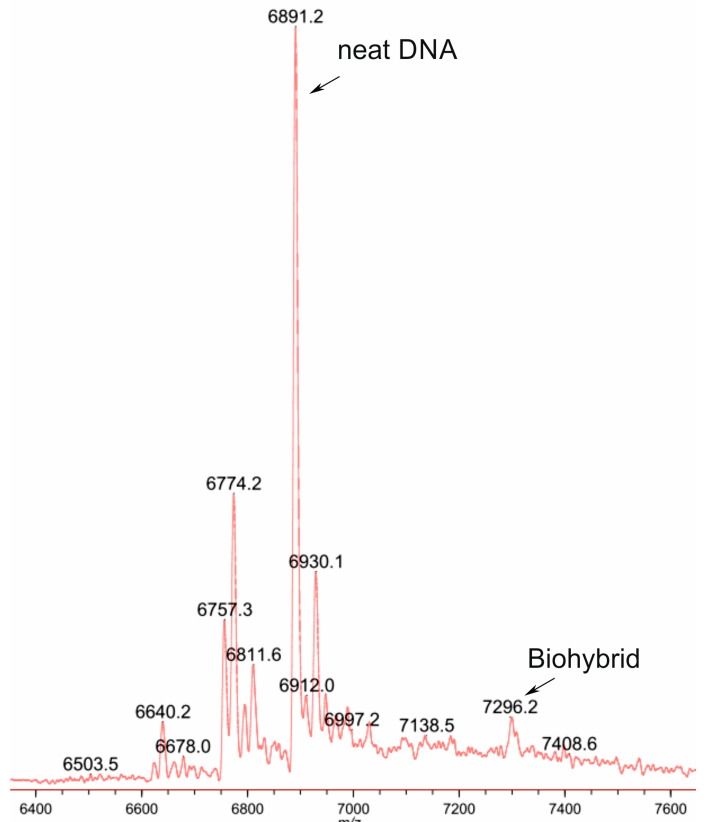
The part of the MALDI-ToF mass spectrum of lyophilized ML showing a neat DNA decamer (*m*/*z* = 6891.2) and an alkylated biohybrid with compound **1** (*m*/*z* = 7296.2).

After the completion of the reaction mixture, the reaction solution was filtered through a membrane; then, the mother liquor (ML, Figure S9) was lyophilized and the MALDI-ToF mass spectrum was obtained. As expected, the MALDI-ToF mass spectrum of the mother liquor, which is presented in Figure 5 (Figure S10), shows peaks that correspond to the free DNA decamer **2B** (*m*/*z* = 6891.2) as a host molecule of a broken molecular complex and an alkylated product (*m*/*z* = 7296.2). 

### 2.10. Topoisomerase Inhibition

Human Topo I is a type I topoisomerase that is able to relax the supercoiled DNA. In order to confirm the influence of the studied compound on the topoisomerase I relaxation activity, we have performed the test that is based upon the measurement of the fluorescence of the DNA stain, which can allow us to differentiate between the supercoiled and relaxed DNA.

**Figure 6:**
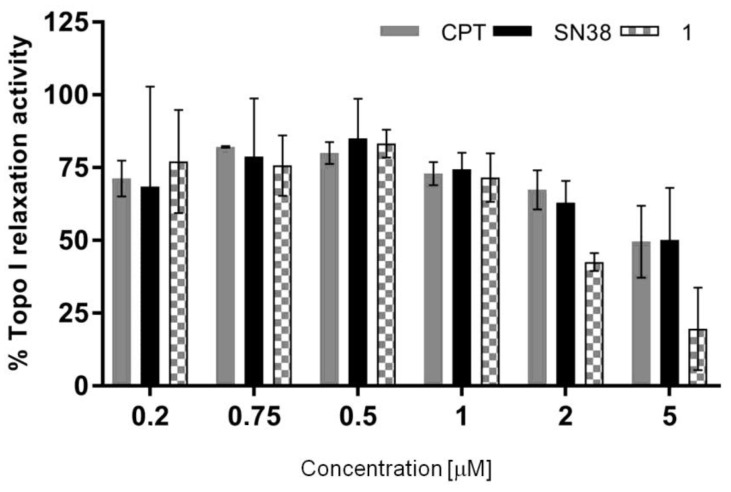
The inhibition of human Topo I relaxation activity with regard to increasing concentrations of CPT, SN38, and new Topo I poison **1**. The mean and SD are presented graphically.

Camptothecin (CPT) and SN38 are well known Topo I interfacial inhibitors, acting as Topo I poisons, and they were chosen as a positive control and reference standard. First, the amount of Topo I that is needed just to fully relax the supercoiled plasmid was determined. Next, we observed a concentration-dependent change in the ratio of the relaxed plasmid after the addition of CPT and SN38 to the reaction mixture containing the DNA plasmid and Topo I (Figure 6). The calculated IC_50_ indices were equal to 11.9 and 7.4 μM (Table 10) for CPT and SN38, respectively. Then, the relaxation activity of human Topo I was measured after the addition of compound **1**. The obtained results show that derivative **1** exhibits a significantly better Topo I relaxation activity than CPT and SN38 do. The IC_50_ index reached the value of 1.7 μM. The presented results confirm that the studied SN38 derivative **1** effectively blocks the DNA relaxation that is mediated by Topo I, which means that it stops the machinery of the Topo I activity.

## 3. Materials and Methods

### 3.1. Chemical Substrates

Nicked decamer **2B** was purchased from FutureSynthesis and purified by filtering on a membrane of 3 kDa. 

The preparation of 7-ethyl-9-[*N*-(2′-hydroxyethyl)amino]methyl-10-hydroxycamptothecin (**1**). SN38 (7-ethyl-10-hydroxycamptothecin × H_2_O) (3.17 mg; 7.7 × 10^−3^ mmol) was suspended in 3.4 mL of CH_3_CN, and then supplemented with acetic acid (98 μL), 37% aqueous CH_2_O (7.5 μL; 0,093 mmol), and 6 μL of ethanolamine (99%) (0.093 mmol). The resulting mixture was stirred at a temperature of 80 °C. After 8 h the solvent was evaporated under a vacuum, and the residue was rinsed with diethyl ether (3 × 1 mL). The residue was purified using HPLC on an RP-C18 LPH column (150 mm × 10 mm) using the following mobile phase system: 20% CH_3_CN/80% aqueous 0.1% HCOOH at a flow rate of 3 mL/min. The course of the chromatography was monitored using UV detection at a wavelength of 260 nm. Fractions were collected (retention time: 6 min) and they were lyophilized. The product was converted to hydrochloride salt using 0.5 % aqueous HCl (3 mL) and it was lyophilized, yielding a pure product (>98%). The yield was 24%.

Spectral data: 7-ethyl-9-[*N*-(2′-hydroxyethyl)amino]methyl-10-hydroxycamptothecin 1.

The results of the HR-MS (ESI) were: when it was calculated for C_25_H_28_N_3_O_6_ [M+H]^+^ = 466.1978, and when it was measured = 466.1975.

The results of the ^1^H NMR δ (D_2_O, pH = 4, TSPA) at 500 MHz were: 0.97 (t, J = 7.3 Hz, 3H), 1.54 (t, J = 7.7 Hz, 3H), 1.98 (m, 2H), 3.20 (m, 2H), 3.36 (bs, 2H), 3.93 (bs, 2H), 4,80 (in D_2_O, 2H), 5.15 (½ AB, J = 18.7 Hz, 1H), 5.19 (½ AB, J = 18.7 Hz, 1H), 5.42 (½ AB, J = 16.4 Hz, 1H), 5.55 (½ AB, J = 16.4 Hz, 1H), 7.32 (s, 1H), 7.42 (d, J = 9.2 Hz,1H), and 7.88 (d, J = 9.2 Hz, 1H).

The results of the ^13^C NMR δ (D_2_O, TSPA), HSQC, and HMBC were: 6.6, 12.9, 25.6, 30.1, 43.9, 48.5, 49.8, 55.9, 65.4, 73.2, 97.7, 109.2, 117.9, 120.7, 127.1, 130.4, 132.4, 144.1, 145.6, 147.2, 150.5, 156.4, 157.6, and 174.4.

The UV values (H_2_O, d = 0.5 cm) were 224 nm, 268 nm, 328 nm, and 378 nm.

The IR values (film in CD_3_CN) were: 1057, 1118, 1166, 1252, 1296,1413, 1455, 1515, 1593, 1657, 1740, 2980, and 3415 cm^−1^.

### 3.2. Sample Preparation

Intermolecular interactions were studied in NMR sample tubes in solutions as described below.

Sample **1** + **2B**: Decamer **2B** (1.95 nmol) was dissolved in 650 µL of NaCl/K_3_PO_4_ buffer, 25 mM each in D_2_O, and 3.9 nmol of compound **1** was added as a solid; pH was adjusted to 6.

### 3.3. NMR Experiments

NMR spectra were recorded at 298 K on a Varian VNMRS-500 spectrometer equipped with a 5 mm Z-SPEC Nalorac IDG 500-5HT gradient probe. The experiments were performed in a buffer solution (25 mM NaCl/25 mM K_3_PO_4_ in D_2_O). The spectra were referenced against TSPA-d_4_. Standard pulse sequences were used.

The experiments were performed under the following conditions:

TOCSY—spectral widths were 5000 Hz in both dimensions, 1024 complex points in t_2_, 320 complex points in t_1_, 80 scans per increment, 1 s relaxation delay, and 90 ms spin-lock time.

NOESY—spectral widths 5000 Hz in both dimensions, 1024 complex points in t_2_, 320 complex points in t_1_, 64 scans per increment, 1 s relaxation delay, and 200 ms mixing time. 

Oneshot [27] DOSY spectra—64 transients, 16 dummy scans, diffusion time (Δ) 250 ms, total diffusion encoding gradient duration (δ) 2 ms, and sixteen values of the diffusion-encoding gradient incremented from 6 to 50 G/cm in such steps that the strength of the next gradient was equal to the previous gradient squared. Processing was carried out using the VARIAN VNMRJ software with the option of correction for spatially nonuniform pulsed field gradients.

### 3.4. MALDI-ToF MS Experiments

Negative ion MALDI mass spectra were recorded on an AXIMA Performance ToF spectrometer (Shimadzu Biotech, Manchester, UK). For ionization/desorption, a nitrogen laser working at a wavelength of 377 nm was applied. The energy of the laser beam was set slightly above the threshold level. Each mass spectrum was accumulated from at least 200 laser pulse cycles and automatically processed using the Launchpad version 2.9.1 software (Shimadzu Biotech, Kyoto, Japan).

A mixture of a 50 mg/mL solution of 3-hydroxypicolinic acid in 50% acetonitrile and 50 mg/mL solution of diammonium hydrogen citrate in deionized water (8:1, v.v.) was used as a matrix.

### 3.5. Calculating Binding Constants from the Diffusion Coefficients

The binding constants (K_a_) of the complexes were estimated by the analysis of the diffusion coefficient of the DNA (2B), ligand (compound **1**), and DNA–ligand complex as a function of the host and guest concentration according to Equations (1) and (2).

(1) DNA+L ↔DNA·L 

(2) $$K_{a}=\frac{\left[\mathrm{DNA}\cdot L\right]}{\left[\mathrm{DNA}\right]\left[L\right]}$$

where the exchange rate between the uncomplexed and complexed species occurs quickly on the NMR timescale, the observed diffusion coefficients (D, (m^2^ s^−1^)) are a weighted average of the diffusion coefficients of the uncomplexed and complexed forms, and where the weighting factors are the relative population sizes of the respective forms. Thus, the observed diffusion coefficients may be expressed as:

(3) $$D_{\mathrm{OBS}-L}={\mathrm{MF}}_{L}D_{L}+\left(1-{\mathrm{MF}}_{L}\right)D_{\left[\mathrm{DNA}\cdot L\right]}\;$$

(4) $$D_{\mathrm{OBS}-\mathrm{DNA}}={\mathrm{MF}}_{\mathrm{DNA}}D_{\mathrm{DNA}}+\left(1-{\mathrm{MF}}_{\mathrm{DNA}}\right)D_{\left[\mathrm{DNA}\cdot L\right]}$$

where D_OBS-L_ and D_OBS-DNA_ are the observed averaged diffusion coefficients for L and DNA, respectively; D_L_ and D_DNA_ are the diffusion coefficients for uncomplexed L and uncomplexed DNA, respectively; MF_L_ and MF_DNA_ are the molar fractions of uncomplexed L and uncomplexed DNA in the solution containing both molecules, respectively; D_[DNA·L]_ is the diffusion coefficient for the complex.

The K_a_ can be also expressed as:

(5) $$K_{a}=\frac{\left[\mathrm{DNA}\cdot L\right]}{\left(C_{\mathrm{DNA}}-\left[\mathrm{DAN}\cdot L\right]\right)\left(C_{L}-\left[\mathrm{DNA}\cdot L\right]\right)}$$

where C_DNA_ and C_L_ are the initial concentrations of DNA and L, respectively.

The unknown complex concentration can be calculated from equations:

(6) $$\left[\mathrm{DNA}\cdot L\right]=\left(1-{\mathrm{MF}}_{\mathrm{DNA}}\right)C_{\mathrm{DNA}}$$

(7) $$\left[\mathrm{DNA}\cdot L\right]=\left(1-{\mathrm{MF}}_{L}\right)C_{L}$$

where the host molecule is much larger than the guest one is, it can be assumed that the diffusion coefficient of the host guest complex is the same as that of the host molecule:

(8) $$D_{\left[\mathrm{DNA}\cdot L\right]}≅D_{\mathrm{OBS}-\mathrm{DNA}}$$

By combining Equations (3), (5), (7) and (8), K_a_ can be determined.

### 3.6. Molecular Dynamics Calculations

The human DNA topoisomerase I complex with camptothecin and 22-base pair DNA duplex (PDB ID: 1T8I and 1K4T) was used as the starting model structure for calculations. The PDB X-Ray structure was carefully inspected and cleaned up, e.g., a non-standard TGP nucleotide was converted to the standard nucleotide with guanine G base, and all of the boundary nucleotides were caped. The modified DNA decamer structure which was previously established by us [28] (PDB ID: **1G1N**) was also used as the model structure for our simplified DNA receptor. The compound **1** structure was manually docked to the nick in the receptor as needed in all of the four possible stacking orientations. Additionally, the two starting orientations of the bulky substituents ((9-CH_2_–NH–CH_2_-CH_2_-OH) and 7-Et) were taken into consideration. This resulted in eight structures each, which were then subjected to molecular dynamics calculations (MD). All of the MD calculations were carried out using the AMBER 14 suite of programs [29]. The electrostatic potential (ESP) charges were obtained for the ligand compounds, the nonstandard phosphate-containing amino acid PTR linker, and the DNA linkers, as needed, by the HF/6-31G* calculations using the Gaussian 09 program [30]. Next, the RESP charges were calculated by charge fitting them using the multi-conformational procedure of the antechamber module implemented in Amber. The missing GAFF force field parameters were obtained using the parmchk module. Each complex was neutralized by adding Na^+^ cations, and then, they were solvated by TIP3 water molecules with a spacing distance of approximately 15 Å around the system surface, thereby creating a periodic box. All of the complexes were subjected to molecular dynamics simulations (MD) using the pmemd.cuda Amber 14 module with the NVIDIA GPU acceleration and mixed ff12SB-GAFF force field. The particle mesh Ewald (PME) [31] method was used to treat long-range electrostatic interactions, and a 10 Å cutoff was applied to the nonbonded Lennard–Jones interactions. The SHAKE algorithm was applied to constrain all of the bonds involving hydrogen atoms, and a 2 fs time step was used in the dynamics simulation. First, the systems were minimized in two stages: the first stage restrained the atomic positions of the solute and only relaxed the water, and the second stage released the restraint and allowed all of the atoms to relax (both with 10,000 minimization steps). Next, the systems were slowly heated to 300 K using an NVT ensemble and 1,000,000 steps using the Langevin dynamics temperature control (gamma_ln = 1.0). Then, the systems were carefully equilibrated at NPT-ensemble simulations at 1 bar pressure with gamma_ln = 5.0. The equilibrations lasted until the system reached a converged density value, which usually lasted for 10–20 ns. Finally, the NPT (constant pressure/temperature) production molecular dynamics were run for 500 or 1000 ns of simulations.

### 3.7. Calculating Binding Free Energies (Enthalpies) Using the MM-PBSA and MM-GBSA Methods [32] 

The MD trajectories were uniformly sampled, thus yielding 25,000 samples for each trajectory. The water and Na^+^ cations were stripped, and the binding free energies (enthalpies) were calculated according to the following equations:

ΔG°_Bind,Solv_ = ΔG°_Bind,Vacuum_ + ΔG°_Solv,Complex_ − (ΔG°_Solv,Ligand_ + ΔG°_Solv,Receptor_) (9)

Solvation free energies were calculated by either solving the linearized Poisson–Boltzmann or generalized Born equation for each of the three states (this provided the electrostatic contribution to the solvation free energy) and adding an empirical term for hydrophobic contributions:

ΔG°_Solv_ = ΔG°_electrostatic,ϵ=80_ − ΔG°_electrostatic,ϵ=1_ + ΔG°_hydrophobic_ (10)

ΔG°_Vacuum_ was obtained by calculating the average interaction energy between receptor and ligand and taking the entropy change that occurred upon binding into account.

ΔG°_Vacuum_ = ΔE°_MM_ − TΔS° (11)

where G°_Bind,Solv_ is the free energy of binding of solvated molecules; G°_Bind,Vacuum_ is the binding free energy in a vacuum; G°_Solv,Complex_, G°_Solv,Ligand_, and G°_Solv,Receptor_ are the solvation free energy for complex, ligand, and receptor molecules, respectively; G°_Electrostatic_ is the electrostatic solvation free energy; G°_Hydrophobic_ is the hydrophobic (nonpolar) solvation free energy; E°_MM_ is the molecular mechanic energy; T is the temperature; S° is the entropy. 

The entropy contribution in our calculations was neglected because of the comparison of states of similar entropy. All of the free energy calculations were carried out using the mm_pbsa.pl script from AmberTools.

### 3.8. PM7 Semi-Empirical Calculations

The MD trajectories were uniformly sampled to yield 5000 structures for each. The water and Na+ cations were stripped, and the DNA was shortened to one GC, and one TA base pair which was complexed with compound **1** on both sides of it, respectively. The remaining DNA base pairs were capped from the cut side with the phosphorane groups. The structures that we prepared were energy minimized with the PM7 method using the MOPAC2016 program [33]. The water solvent was approximated with the COSMO model [34]. 

### 3.9. Cluster Analysis

The CPPTRAJ module implemented in the Amber package was used for cluster analysis. During cluster analysis, similar conformations were identified and grouped together. During clustering analysis, the K-means clustering algorithm was used. The RMSD of heavy atoms was used as a distance metric which were calculated only for the ligand derivative and the neighboring two DNA base pairs on each side for the structures from PBSA/GBSA calculations and one DNA base pair for structures from PM7 calculations. The clustering procedure was repeated several times, and each time the low-population strange structures were gradually removed. Finally, for each system, the most populated clusters were obtained. For each cluster, the average energies were calculated (PBSA/GBSA or PM7), and the most representative structures were determined.

### 3.10. Docking Calculations

Docking calculations were performed using the AutoDock suite of programs [35]. AutoDock is a computational docking program that is based on the empirical free energy force field and rapid Lamarckian genetic algorithm search method [36,37]. To overcome the rigid receptor simplification in AutoDock, docking studies were performed on an ensembles of structures of complexes which were obtained from molecular dynamic simulations. For each complex, the set of 1000 uniformly sampled structures were used. The coordinate files were prepared using AutoDockTools, and pre-calculations of atomic affinities were performed using AutoGrid. Both of the programs are parts of AutoDock suite. The standard docking parameters were used together with the GPU-accelerated version of AutoDock4.2.6. The fifty possible binding conformations were generated each time and analyzed, and they were sorted by family structures using home build scripts.

### 3.11. The Hydrogen Bonds Analysis

Hydrogen bonds were identified and analyzed using proprietary scripts and the UCSF Chimera version 1.14 program [38]. The chimera program was also used to visualize chemical structures. Table 4 lists only the hydrogen bonds with a population greater than 10%, at least for one of the calculation methods.

### 3.12. The Topo I Relaxation Activity Assay

The inhibition of topoisomerase I relaxation activity was tested in triplicate using the Human Topoisomerase I Relaxation High-Throughput Plate Assay (Inspiralis Ltd., Norwich, UK) according to the vendor’s protocol. Briefly, the test is based upon the measurement of fluorescence of a DNA stain which can be used to differentiate between supercoiled and relaxed DNA. The inhibition of human Topo I relaxation activity was measured in an assay mixture containing Topo I and a supercoiled DNA substrate (plasmid pNO1). In the first step, the amounts of enzyme that is needed to fully relax the plasmid was determined. Next, for this specific amount of enzyme, the relaxation inhibition potency of the studied compound was tested. The increasing amount of compounds (0.2–5 μM) were added to the reaction mixture together with positive controls and reference standards: CPT and SN38, respectively. The fluorescence of DNA plasmid that was stained with Diamond™ Nucleic Acid Dye (Promega, Madison, WI, USA) was read in a fluorescence plate reader TECAN Infinite M1000 PRO (495 nm/537 nm) [39,40].

## 4. Conclusions

Compound **1** has been studied using a computational docking and molecular dynamic free energy approach as a ligand in a binary DNA/Topo I receptor that forms a ternary complex, which makes it a biologically relevant target for Topo I inhibitors. The calculation data that were obtained clearly favors compound **1** in terms of the DNA/Topo I receptor binding strength over the known Topo I inhibitors such as CPT, SN38, and TPT. This appears to be due to the unique ability of the C-9 hydroxyalkylamino substituent in compound **1** to form hydrogen bonds with both the DNA and topoisomerase I. In addition, the calculations indicate that compound **1** may bind the DNA/Topo I complex (**2A**) in two different orientations. One is identical to those which were observed in the X-ray structures of the CPT/DNA/Topo I and TPT/DNA/Topo I ternary complexes, and the other is slightly preferred, wherein compound **1** is rotated 180° degrees about an axis that is perpendicular to the plane of the molecule (Figure 1). In the case of the binary complex **1**/DNA, both the experimental, which was NOESY-derived, and computational data indicate that the second possible orientation is that in which derivative **1** is rotated 180° degrees about a long axis that is parallel to the artificial plane of its molecule (Figure 4). The other relevant experimental techniques, DOSY-NMR and MALDI-ToF MS, provide confirmation that compound **1** not only creates a very strong complex with **2B** due to the ability of the C-9 hydroxyalkylamino substituent to form hydrogen bonds with the DNA (**2B**), but it also has the capability to alkylate the DNA. Moreover, the presented experimental and computational data are in agreement with the results of the Topo I relaxation activity test, showing that compound **1** inhibits the Topo I relaxation activity more efficiently than CPT and SN38 do.

## Figures and Tables

**Table 1:** Docking analysis with AutoDock-GPU.

Ligand	Calculated Free Energy of Binding FE [kcal/mol] and Inhibition Constant Ki [nM]							
	X-ray Single Structure				1000 MD-Derived Structures			
	1K4T		1T8I		1K4T		1T8I	
	FE	Ki	FE	Ki	FE	Ki	FE	Ki
Compound **1**	−12.15	1.24	−11.59	3.22	−10.98 ± 0.57	8.92	−11.88 ± 0.88	1.95
TPT	−11.81	2.22	−11.47	3.89	−10.15 ± 0.49	36.25	−10.96 ± 0.79	9.22
CPT	−11.57	3.29	−11.20	6.19	−10.38 ± 0.47	24.48	−10.95 ± 0.77	9.38
SN38	−11.26	5.57	−10.69	14.61	−10.36 ± 0.48	25.42	−11.04 ± 0.76	8.08

**Table 2:** Docking analysis for receptor structures obtained in MD simulations of ligand/DNA/Topo I ternary complex.

Ligand	Calculated Free Energy of Binding [kcal/mol], Inhibition Constant Ki [nM], and Cluster Size CS											
	Family Structure 1			Family Structure 2			Family Structure 3			Family Structure 4		
	Energy	Ki	CS	Energy	Ki	CS	Energy	Ki	CS	Energy	Ki	CS
**Compound 1**	−10.97 ± 0.46	9.17	1883	−11.27 ± 0.75	5.46	2125	−10.73 ± 0.06	13.56	82	−10.65 ± 0.04	15.50	63
**TPT**	−10.27 ± 0.40	23.60	1956	−10.49 ± 0.47	25.36	1196	−10.04 ± 0.01	44.12	11	−9.98 ± 0.03	48.44	93
**CPT**	−10.33 ± 0.41	27.03	2844	−10.06 ± 0.05	42.20	168	−9.96 ± 0.01	50.49	22	−10.01 ± 0.01	46.19	19
**SN38**	−10.38 ± 0.41	24.54	2407	−10.40 ± 0.35	27.79	1219	−10.25 ± 0.10	30.80	203	−10.04 ± 0.02	44.04	36

**Table 3:** MM-PBSA and MM-GBSA results of calculations for ligand/DNA/Topo I ternary complex.

Ligand/Family Structure	Free Energy of Binding [kcal/mol]					
	MM-PBSA			MM-GBSA		
	Trajectory A	Trajectory B	(A + B)/2	Trajectory A	Trajectory B	(A + B)/2
Compound **1**/**1**	−42.19 ± 2.03	−38.02 ± 3.46	−40.10 ± 2.75	−51.81 ± 1.99	−51.75 ± 2.01	−51.78 ± 2.00
Compound **1**/**2**	−33.37 ± 1.71	−41.32 ± 4.84	−37.35 ± 3.28	−38.80 ± 1.68	−51.53 ± 5.34	−45.16 ± 3.51
Compound **1**/**3**	−32.46 ± 3.02	−34.34 ± 1.49	−33.40 ± 2.36	−39.34 ± 3.93	−39.60 ± 2.62	−39.47 ± 3.28
Compound **1**/**4**	−22.94 ± 2.38	−30.85 ± 1.50	−26.89 ± 1.94	−33.21 ± 1.29	−38.60 ± 2.48	−35.91 ± 1.89
TPT/1	−39.21 ± 1.62			−48.03 ± 0.58		
CPT/1	−38.69 ± 3.81			−48.89 ± 3.73		

**Table 4:** Hydrogen bond (HB) statistic for compound **1**/DNA/Topo I ternary complex (only HB between compound **1** and DNA/Topo I receptor are reported).

Atom Names		Docking Family Structure 1		MD Family Structure 1	
Receptor	Compound 1	HB Length [Å]	Population	HB Length [Å]	Population
DA 35 H62	9-CH2NHC2H4**O**H	1.99 ± 0.18	57.39%	2.69 ± 0.63	1.20%
DA 35 N7	9-CH2NHC2H4O**H**	2.06 ± 0.12	50.85%	-	-
DA 35 N7	9-CH2N**H**C2H4OH	2.35 ± 0.29	48.65%	2.67 ± 0.88	0.50%
LYS 376 HZ1, 2, 3	21 >C=**O**	2.34 ± 0.45	48.50%	2.10 ± 0.43	75.55%
ARG 208 HH12	1-**N**	2.47 ± 0.36	28.73%	2.87 ± 0.32	39.80%
ASP 377 OD1, 2	20 -O**H**	2.13 ± 0.44	27.85%	2.47 ± 0.79	59.05%
LYS 269 HZ1, 2, 3	9-CH2NHC2H4**O**H	2.02 ± 0.37	20.73%	2.24 ± 0.53	4.90%
DA 35 OP2	10 -O**H**	2.52 ± 0.35	19.65%	-	-
DT 9 O4	9-CH2NHC2H4O**H**	2.10 ± 0.23	19.00%	2.29 ± 0.89	0.75%
TYR 270 O	9-CH2NHC2H4O**H**	2.07 ± 0.20	16.50%	1.88 ± 0.37	23.20%
TYR 270 H	9-CH2NHC2H4**O**H	2.00 ± 0.21	12.62%	2.25 ± 0.35	11.25%
DG5 11 HO5′	16 >=**O**	2.72 ± 0.58	8.50%	2.67 ± 0.68	11.50%
DC 34 H42	9-CH2NHC2H4**O**H	2.05 ± 0.38	1.50%	2.18 ± 0.34	34.55%
ASN 196 HD22	9-CH2NHC2H4**O**H	2.09 ± 0.40	1.54%	2.16 ± 0.33	13.05%
Average H-bond number per residue in family structure 1			**3.62**		**2.75**
		Docking Family Structure 2		MD Family Structure 2	
ARG 208 HH11, 12	16 >C=**O**	2.57 ± 0.67	59.57%	2.51 ± 0.71	29.45%
LYS 376 HZ1, 2, 3	9-CH2NHC2H4**O**H	2.00 ± 0.24	55.30%	2.17 ± 0.43	34.65%
ASN 566 OD1	10 -O**H**	2.02 ± 0.20	45.13%	1.84 ± 0.20	61.00%
DT3 10 O2	9-CH2NHC2H4O**H**	1.99 ± 0.13	44.70%	2.28 ± 0.46	7.05%
LYS 269 HZ1, 2, 3	21 >C=**O**	2.31 ± 0.52	42.35%	2.58 ± 0.58	34.60%
ASN 566 HD21	10 -**O**H	2.17 ± 0.26	39.87%	2.09 ± 0.20	35.15%
DA 35 H62	20 -**O**H	2.98 ± 0.29	29.48%	2.99 ± 0.32	30.90%
THR 562 OG1	9-CH2N**H**C2H4OH	2.12 ± 0.21	29.30%	2.47 ± 0.35	16.75%
LYS 269 HZ1, 2, 3	21 >**O**	2.57 ± 0.58	28.39%	2.44 ± 0.42	22.80%
DT3 10 O3′	9-CH2NHC2H4O**H**	2.09 ± 0.29	17.70%	2.45 ± 0.55	4.35%
DG5 11 O3′	9-CH2NHC2H4O**H**	2.00 ± 0.23	15.17%	2.26 ± 0.60	3.85%
DG5 11 H21	16 >=**O**	3.33 ± 0.35	14.04%	3.01 ± 0.85	0.75%
DG5 11 O4′	9-CH2N**H**C2H4OH	2.02 ± 0.21	13.74%	2.56 ± 0.66	1.05%
DT3 10 O2	9-CH2N**H**C2H4OH	2.39 ± 0.31	11.61%	2.50 ± 0.28	34.30%
THR 562 OG1	10 -O**H**	2.13 ± 0.21	11.04%	2.79 ± 0.40	8.75%
ASP 377 OD1, 2	9-CH2NHC2H4O**H**	2.28 ± 0.55	10.65%	2.41 ± 0.29	18.65%
DA 35 N6	20 -O**H**	-	-	3.14 +/−0.27	23.900
Average H-bond number per residue in family structure 2			**4.68**		**3.68**
		Docking Family Structure 3		MD Family Structure 3	
DA 35 O4′	10 -O**H**	2.13 ± 0.34	39.54%	2.39 ± 1.69	0.10%
ARG 208 HH12, 22	16 >C=**O**	2.22 ± 0.75	27.13%	2.34 ± 0.58	97.80%
DC 34 O2	9-CH2N**H**C2H4OH	2.07 ± 0.38	25.58%	-	-
DC 34 O3′	9-CH2NHC2H4O**H**	2.11 ± 0.58	16.28%	2.80 ± 2.01	0.10%
TYR 270 O	10 -O**H**	2.03 ± 0.50	15.50%	1.82 ± 0.20	22.00%
DC 34 O4′	9-CH2NHC2H4O**H**	2.02 ± 0.54	13.95%	-	-
DC 34 OP2	9-CH2NHC2H4O**H**	2.16 ± 0.65	13.95%	1.80 ± 0.25	18.20%
ASN 566 HD21	16 >=**O**	2.56 ± 0.91	12.40%	-	-
DA 35 O4′	9-CH2N**H**C2H4OH	2.48 ± 0.72	11.63%	-	-
DG5 11 N7	20 -O**H**	3.05 ± 0.93	9.30%	2.12 ± 0.33	43.70%
DG5 11 HO5′	21 >C=**O**	2.24 ± 0.79	8.53%	2.34 ± 0.54	19.15%
DG5 11 O4′	20 -O**H**	2.40 ± 1.01	6.20%	2.20 ± 0.48	17.30%
GLU 200 H	9-CH2NHC2H4**O**H	1.87 ± 0.71	5.43%	2.28 ± 0.29	32.00%
ASN 566 HD21	21 >**O**	2.06 ± 0.93	3.88%	2.86 ± 0.44	22.05%
Average H-bond number per residue in family structure 3			**2.09**		**2.73**
		Docking Family Structure 4		MD Family Structure 4	
ASN 566 OD1	9-CH2NHC2H4O**H**	2.05 ± 0.43	42.68%	1.79 ± 0.21	14.60%
ASN 196 HD22	21 >C=**O**	2.17 ± 0.50	30.49%	2.28 ± 0.34	19.95%
ASN 196 HD22	21 >**O**	2.55 ± 0.69	29.27%	2.41 ± 0.41	20.00%
DC 34 O3′	20 -O**H**	2.66 ± 0.77	25.61%	2.63 ± 0.60	3.15%
DG5 11 O4′	9-CH2N**H**C2H4OH	2.04 ± 0.55	18.29%	2.36 ± 0.34	15.75%
ASN 566 HD21	9-CH2NHC2H4**O**H	2.11 ± 0.63	18.29%	2.50 ± 0.52	1.70%
ASN 566 OD1	9-CH2N**H**C2H4OH	2.13 ± 0.60	15.85%	-	-
ASN 566 HD21	10 -**O**H	1.98 ± 0.70	14.63%	-	-
THR 562 OG1	9-CH2N**H**C2H4OH	2.05 ± 0.67	13.42%	2.86 ± 1.09	0.35%
DA 35 OP2	20 -O**H**	2.38 ± 0.78	13.42%	2.80 ± 0.41	5.45%
ASN 566 OD1	10 -O**H**	2.07 ± 0.67	12.20%	-	-
DG5 11 HO5′	10 -**O**H	2.00 ± 0.75	12.20%	2.16 ± 0.40	4.65%
LYS 218 HZ1, 2, 3	20 -**O**H	2.25 ± 1.53	10.98%	1.94 ± 0.29	47.30%
GLU 200 H	16 >C=**O**	2.01 ± 0.76	9.76%	2.25 ± 0.26	49.65%
DA 35 N6	9-CH2N**H**C2H4OH	-	-	2.45 ± 0.31	23.60%
DT3 10 O4	9-CH2NHC2H4O**H**	-	-	1.96 ± 0.38	18.00%
Average H-bond number per residue in family structure 4			**2.67**		**2.24**

**Table 5:** The ^1^H NMR chemical shift changes in DNA (**2B**). Please also refer to Table S2.

Base	H1′	H2′	H2″	H3′	H4′	H5′, H5″	H6/8	H2/5/Me
T4	0.00	−0.013	−0.061	0.012	−0.041	-	0	−0.022
T5	0.01	0.045	−0.062	−0.005	−0.022	-	0.069	−0.020
nick	-----------	----------	----------	-----------	----------	------------------	-----------	-----------
G6	−0.36	−0.326	−0.081	0.02	−0.053	−0.08/−0.26	0.009	-
T7	−0.06	−0.029	−0.051	−0.025	−0.115	-	−0.236	−0.027
								
A14	−0.02	0.009	−0.15	−0.122	−0.059	-	−0.094	−0.06
C15	−0.09	0.238	0.142	-	0.02	-	0.035	−0.265
A16	0.057	0.020	−0.107	−0.045	-	-	−0.247	−0.218
A17	−0.02	−0.056	−0.045	−0.034	−0.029	-	−0.088	−0.101

Red is used to highlight the protons involved in intermolecular cross-peaks.

**Table 6:** MM-PBSA and MM-GBSA results of calculations for compound **1**/DNA binary complex.

Family Structure	Free Energy of Binding [kcal/mol]					
	MM-PBSA			MM-GBSA		
	Trajectory A	Trajectory B	(A + B)/2	Trajectory A	Trajectory B	(A + B)/2
1	−32.29 ± 4.35	−32.23 ± 4.34	−32.26 ± 4.40	−33.09 ± 3.17	−32.71 ± 3.33	−32.91 ± 3.28
2	−32.99 ± 3.41	−27.10 ± 5.12	−30.05 ± 4.56	−30.63 ± 2.86	−24.76 ± 4.76	−27.69 ± 3.85
3	−37.52 ± 3.47	−35.74 ± 3.80	−36.63 ± 3.93	−32.80 ± 2.99	−31.46 ± 3.03	−32.13 ± 3.16
4	−34.04 ± 3.05	−34.20 ± 3.15	−34.12 ± 3.12	−33.44 ± 2.37	−33.45 ± 2.44	−33.45 ± 2.40

**Table 7:** PM7 semi-empirical results of calculations for compound **1**/DNA binary complex.

Family Structure	PM7 Energy [kcal/mol]		
	Trajectory A	Trajectory B	(A + B)/2
1	−2378.18 ± 14.84	−2378.04 ± 16.51	−2378.11 ± 15.69
2	−2379.80 ± 18.12	−2378.37 ± 23.54	−2379.09 ± 20.88
3	−2385.59 ± 16.02	−2384.05 ± 21.30	−2384.82 ± 20.60
4	−2376.71 ± 19.07	−2377.87 ± 15.68	−2377.29 ± 15.62

**Table 8:** Comparison of measured distance statistics for selected protons in conformers recorded during MD trajectories for four possible family structures with observed intermolecular NOE effects.

Comp. 1	DNA	NOE ^a^	Family Structure 1 ^b^				Family Structure 2				Family Structure 3				Family Structure 4			
			<6 Å	<5 Å	<4 Å	<3 Å	<6 Å	<5 Å	<4 Å	<3 Å	<6 Å	<5 Å	<4 Å	<3 Å	<6 Å	<5 Å	<4 Å	<3 Å
14	A16H2	68%	++++	++++	++++	+++	++	o							++++	++++	++++	+
17a	G6H2″	90%	++++	+++	+	o					+++							
17b			++++	o	o						++++	+++	+					
17a	G6H4′	75%	++++	++++	++++	+++					++	+						
17b			++++	++++	++++	++					+++	++	++	o				
17a	T5H1′	70%	++++	++							+	o						
17b			++++	++++	++						+	o						
19	G6H2″	14%	++	+	o													
19	G6H4′	45%	+++	++	+													
19	T7H4′	43%	++++	++++	++++	+									o			
19	T7H5′	60%	++++	+++	+++	+									o			
19	T7H6	18%	++															
23	C15H6	8%	o															

^a^ Percentage of NOE effect compared to NOE between cytidine H5/H6 protons. ^b^ ++++, +++, ++, +, and o represent >50%, >25%, >10%, >5%, and >1% MD sampled conformers for a given structure that meet the corresponding distance condition between selected protons, respectively.

**Table 9:** The diffusion constants Di × 10^−10^ (m^2^ s^−1^) and binding constant calculation of a complex **1** + **2B** from DOSY data.

Sample	Concentration (mM)		1 (19-CH_3_) Integral	2B (T7-CH_3_) Integral	D*_i_* of 1 (10^−10^ m^2^ s^−1^)	D*_i_* of 2B (10^−10^ m^2^ s^−1^)	K_a_ (mM^−1^)	% of Complex
	1	2B						
Complex	0.33 ^b^	0.33 ^b^	1	1	1.23	1.13	746.0	94% 6%
Compound **1**	0.7 ^a^	-	-	-	2.75	-	-	-
Decamer **2B**	-	0.96 ^a^	-	-	-	1.13	-	-

^a^ Concentration of compound **1** calculated from the weighted amount. ^b^ Concentration of DNA **2B** calculated from the integral of 19-CH_3_ and T7-CH_3_ signals.

**Table 10:** The IC_50_ indices—a concentration required to reduce the relaxation Topo I activity by 50%. Values are presented as mean ± SD (*n* = 3).

Compound	CPT	SN38	1
IC_50_ [µM] (mean ± SD)	11.92 ± 9.77	7.37 ± 7.10	1.73 ± 0.48

## Data Availability

The authors confirm that the data supporting the findings of this study are available within the article and its Supplementary Materials.

## Supplementary Materials

The following supporting information can be downloaded at: https://www.mdpi.com/article/10.3390/molecules27206946/s1: Figure S1: The ^1^H NMR spectrum of nicked decamer 2B; Figure S2: The ^1^H NMR spectrum of compound 1; Figure S3: NOESY spectrum of sample 1 + 2B; Figure S4: TOCSY spectrum of sample 1 + 2B; Figure S5: The example of the cross-peaks in sample 1+2B; Figure S6: The hydrogen bonding in structures best representing the most populated cluster in complex 1 + 2A; Figure S7: The DOSY spectrum presenting a mixture of compound 1 with 2B at the start of the reaction; Figure S8: The full DOSY spectrum of a 1:1 complex of compound 1 and 2B; Figure S9: The partial DOSY spectrum of a 1:1 complex of compound 1 and 2B; Figure S10: 1D NMR spectrum of reaction mixture of 2B with compound 1 at the start and in a mother liquor (ML); Figure S11: The MALDI-ToF mass spectrum of ML 1 + 2B. Table S1: The ^1^H NMR chemical shifts of DNA 2B with compound 1; Table S2: Chemical shift changes in the DNA decamer; Table S3: The ^1^H NMR chemical shift changes in SN38 derivative 1 after addition of it to DNA.

## Sample Availability

Samples of compound **1** are available from the authors on request.

## References

[1] Han S., Lim K.S., Blackburn B.J., Yun J., Putnam C.W., Bull D.A., Won Y.W. (2022). The Potential of Topoisomerase Inhibitor-Based Antibody-Drug Conjugates. Pharmaceutics.

[2] Staker B.L., Hjerrild K., Feese M.D., Behnke C.A., Burgin A.B., Stewart L. (2002). The mechanism of topoisomerase I poisoning by a camptothecin analog. Proc. Natl. Acad. Sci. USA.

[3] Hevenern K.E., Verstak T.A., Lutat K.E., Riggsbee D.L., Mooney J.W. (2018). Recent developments in topoisomerase-targeted cancer chemotherapy. Acta Pharm. Sin. B.

[4] Stewart L., Redinbo M.R., Qiu X., Hol W.G., Champoux J.J. (1998). A model for the mechanism of human topoisomerase I. Science.

[5] Dallavalle S., Merlini L., Fattorusso E., Taglialatela-Scafati O. (2007). Camptothecin and Analogs: Structure and Synthetic Efforts. Modern Alkaloids.

[6] Liu Y.-Q., Li W.-Q., Morris-Natschke S.L., Qian K., Yang L., Zhu G.-X., Wu X.-B., Chen A.-L., Zhang S.-Y., Nan X. (2015). Perspectives on biologically active camptothecin derivatives. Med. Res. Rev..

[7] Verma R.P., Hansch C. (2009). Camptothecins: A SAR/QSAR study. Chem. Rev..

[8] Redinbo M.R., Stewart L., Kuhn P., Champoux J.J., Hol W.G. (1998). Crystal structures of human topoisomerase I in covalent and noncovalent complexes with DNA. Science.

[9] Bocian W., Kawęcki R., Bednarek E., Sitkowski J., Williamson M.P., Hansen P.E., Kozerski L. (2008). Binding of Topotecan to a Nicked DNA Oligomer in Solution. Chemistry.

[10] Pommier Y., Marchand C. (2011). Interfacial inhibitors: Targeting macromolecular complexes. Nat. Rev. Drug Discov..

[11] Coletta A., Desideri A. (2013). Role of a protein in the DNA sequence specificity of the cleavage site stabilized by the camptothecin topoisomerase IB inhibitor: A metadynamic study. Nucleic Acids Res..

[12] Naumczuk B., Hyz K., Kawęcki R., Bocian W., Bednarek E., Sitkowski J., Wielgus E., Kozerski L. (2015). DOSY NMR and MALDI-TOF evidence of covalent binding the DNA duplex by trimethylammonium salts of topotecan upon near UV irradiation. Magn. Reson. Chem..

[13] Naumczuk B., Kawęcki R., Sitkowski J., Bocian W., Bednarek E., Kozerski L. (2016). Spontaneous 2′-deoxyguanosine alkylation by a new generation of topoisomerase I inhibitors of the camptothecin family. New J. Chem..

[14] Naumczuk B., Kawęcki R., Bocian W., Bednarek E., Sitkowski J., Kozerski L. (2019). Regioselective alkylation reaction of the 2′deoxycitidine with 9-aminomethyl derivatives of SN38. J. Mol. Struct..

[15] Naumczuk B., Bocian W., Sitkowski J., Kawęcki R., Kozerski L. (2019). Solvent-dependent regioselectivity of 2′-deoxyadenosine alkylation by 9-aminomethyl derivatives of SN38. New J. Chem..

[16] Naumczuk B., Wiktorska K., Lubelska K., Kawęcki R., Bocian W., Bednarek E., Sitkowski J., Chilmonczyk Z., Kozerski L. (2016). New generation of camptothecin derivatives spontaneously alkylating DNA. New J. Chem..

[17] Naumczuk B., Górecki M., Wiktorska K., Urbanowicz M., Sitkowski J., Lubelska K., Milczrek M., Bednarek E., Bocian W., Kozerski L. (2021). New camptothecin derivatives for generalized oncological chemotherapy: Synthesis, stereochemistry and biology. Bioorg. Med. Chem. Lett..

[18] Naumczuk B., Sitkowski J., Bocian W., Stolarska M., Bednarek E., Kozerski L. (2021). A NMR Study of Binding the Metabolite of SN38 derivatives to a Model Nicked DNA Decamer Mimicking Target of Topo I Inhibitors. Bioorg. Chem..

[19] Bocian W., Naumczuk B., Urbanowicz M., Sitkowski J., Bierczyńska-Krzysik A., Bednarek E., Wiktorska K., Milczrek M., Kozerski L. (2021). The Mode of SN38 Derivatives Interacting with Nicked DNA Mimics Biological Targeting of Topo I Poisons. Int. J. Mol. Sci..

[20] Santos-Martins D., Solis-Vasquez L., Tillack A.F., Sanner M.F., Koch A., Forli S. (2021). Accelerating AutoDock4 with GPUs and Gradient-Based Local Search. J. Chem. Theory Comput..

[21] Staker B.L., Feese M.D., Cushman M., Pommier Y., Zembower D., Stewart L., Burgin A.B. (2005). Structures of three classes of anticancer agents bound to the human topoisomerase I-DNA covalent complex. J. Med. Chem..

[22] Ruvinsky A.M., Kozintsev A.V. (2005). New and fast statistical-thermodynamic method for computation of protein-ligand binding entropy substantially improves docking accuracy. J. Comput. Chem..

[23] Chen D., Oezguen N., Urvil P., Ferguson C., Dann S.M., Savidge T.C. (2016). Regulation of protein-ligand binding affinity by hydrogen bond pairing. Sci. Adv..

[24] Williamson M.P. (2009). Applications of the NOE in Molecular Biology. Annual Report NMR Spectroscopy.

[25] Antałek B. (2002). Using Pulsed Gradient Spin Echo NMR for Chemical Mixture Analysis: How to Obtain Optimum Results. Concepts Magn. Reson..

[26] Antałek B. (2006). Accounting for spin relaxation in quantitative pulse gradient spin echo NMR mixture analysis. J. Am. Chem. Soc..

[27] Pelta M.D., Morris G.A., Stchedroff M.J., Hammond S.J. (2002). A one-shot sequence for high-resolution diffusion-ordered spectroscopy. Magn. Reson. Chem..

[28] Kozerski L., Mazurek A.P., Kawęcki R., Bocian W., Krajewski P., Bednarek E., Sitkowski J., Williamson M.P., Moir A.J., Hansen P.E. (2001). A nicked duplex decamer DNA with a PEG(6) tether. Nucleic Acids Res..

[29] Case D.A., Berryman J.T., Betz R.M., Cerutti D.S., Cheatham III T.E., Darden T.A., Duke R.E., Giese T.J., Gohlke H., Goetz A.W. (2015). Amber 2015.

[30] Frisch J., Trucks G.W., Schlegel H.B., Scuseria G.E., Robb M.A., Cheeseman J.R., Scalmani G., Barone V., Mennucci B., Petersson G.A. (2009). Gaussian 09, Revision A.01.

[31] Essmann U., Perera L., Berkowitz M.L., Darden T., Lee H., Pedersen L.G. (1995). A Smooth Particle Mesh Ewald Method. J. Chem. Phys..

[32] Luo R., David L., Gilson M.K. (2002). Accelerated Poisson-Boltzmann calculations for static and dynamic systems. J. Comput. Chem..

[33] Stewart J. (2016). Mopac2016.

[34] Klamt A., Schuurmann G. (1993). Cosmo—A New Approach to Dielectric Screening in Solvents with Explicit Expressions for the Screening Energy and Its Gradient. J. Chem. Soc. Perkin Trans..

[35] Forli S., Huey R., Pique M.E., Sanner M.F., Goodsell D.S., Olson A.J. (2016). Computational protein-ligand docking and virtual drug screening with the AutoDock suite. Nat. Protoc..

[36] Goodsell D.S., Olson A.J. (1990). Automated docking of substrates to proteins by simulated annealing. Proteins.

[37] Morris G.M., Goodsell D.S., Halliday R.S., Huey R., Hart W.E., Belew R.K., Olson A.J. (1998). Automated docking using a Lamarckian genetic algorithm and an empirical binding free energy function. J. Comput. Chem..

[38] Pettersen E.F., Goddard T.D., Huang C.C., Couch G.S., Greenblatt D.M., Meng E.C., Ferrin T.E. (2004). UCSF chimera—A visualization system for exploratory research and analysis. J. Comput. Chem..

[39] Maxwell A., Burton N.P., O’Hagan N. (2006). High-throughput assays for DNA gyrase and other topoisomerases. Nucleic Acids Res..

[40] Seol Y., Zhang H., Agama K., Lorence N., Pommier Y., Neuman K.C. (2015). Single-Molecule Supercoil Relaxation Assay as a Screening Tool to Determine the Mechanism and Efficacy of Human Topoisomerase IB Inhibitors. Mol. Cancer Ther..

