# Supplementary material for: Insight on the Interaction between the Camptothecin Derivative and DNA Oligomer Mimicking the Target of Topo I Inhibitors
Source: Molecules. 2022 Oct 17;27(20):6946. doi: 10.3390/molecules27206946 (PMC9612166; doi:10.3390/molecules27206946)
Supplement: Supplementary file 1 [file molecules-27-06946-s001.zip › molecules-1934993-supplementary.pdf]

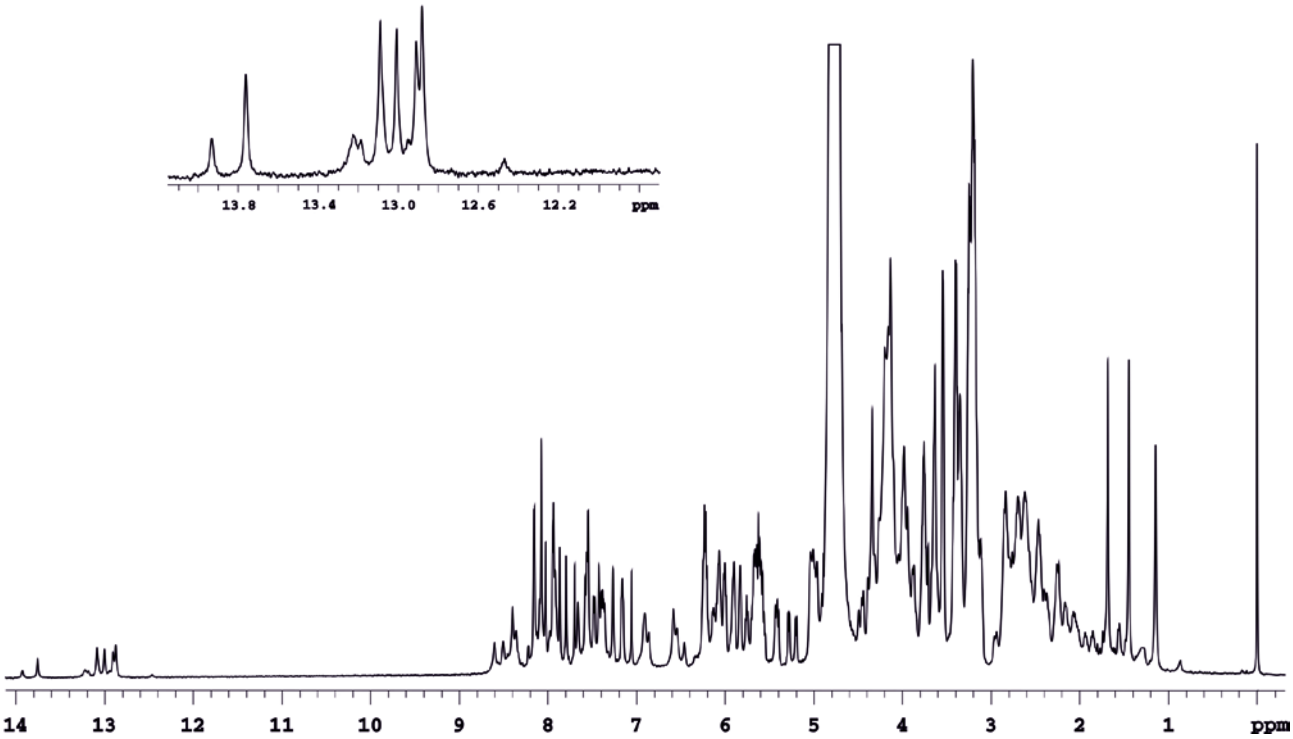

Figure S1 The ^{1}H NMR spectrum of nicked decamer **2B** in buffered H_2O/D_2O. (90/10 vol%) 25 mM NaCl /25 mM K_3PO_4. at pH 6. Seven guanosine and 3 tymidine NHs hydrogen bonded. forming a duplex are shown in an inset.

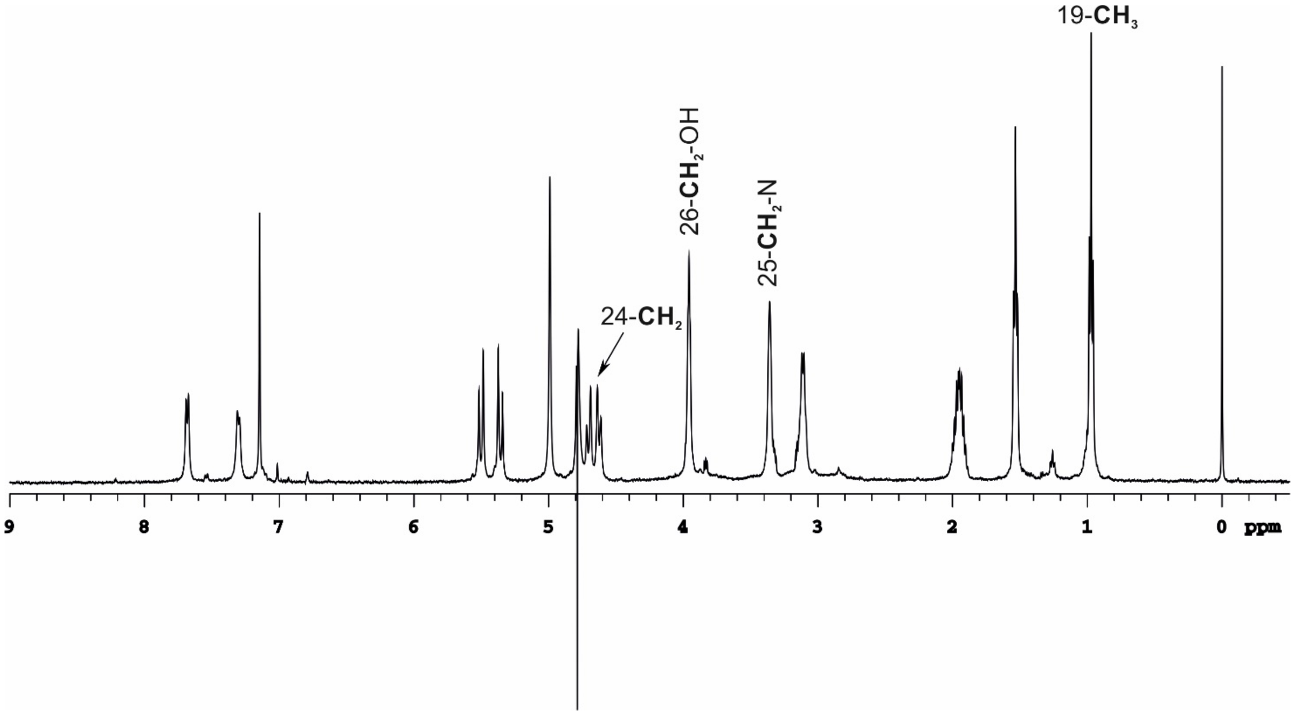

Figure S2 The ^{1}H NMR spectrum of **1** in buffered /D_2O. 25 mM NaCl /25 mM K_3PO_4. at pH 6.

Table S1. The ^{1}H NMR chemical shifts δ [ppm] of DNA **2B** and SN38 derivative **1** from NOESY spectrum in buffered D_2O. at 25°C **(1:3** ratio) *

Base	H1'	H2'	H2"	H3'	H4'	H5'. H5"	H6/8	H2/5/Me
G1	5.993	2.772	2.833	4.958	4.332	3.879/3.987	8.058	-
C2	5.694	2.125	2.426	4.861	4.208	na/4.163	7.377	5.372
G3	6.046	2.672	2.806	5.002	4.422	na/ na	7.907	-
T4	6.06	2.071	2.546	4.883	4.233	na	7.268	1.424
**T5**	**6.215**	**2.543**	**2.555**	**4.985**	**4.178**	**na**	7.494	**1.671**
nick	-----------	-----------	-----------	-----------	-----------	-----------------------	-----------	-----------
**G6**	**5.47**	**2.316**	**2.561**	**4.69**	**4.154**	**3.794/ 3.873**	**7.674**	-
T7	6.006	2.151	2.506	4.846	4.202	na/ 4.108	7.306	1.120
C8	5.725	2.002	2.355	4.838	4.09	na/ na	7.427	5.587
G9	5.896	2.623	2.69	4.999	4.353	na/ na	7.917	-
C10	6.219	2.234	2.463	4.844	4.188	na / 4.245	7.56	5.594

Base	H1'	H2'	H2"	H3'	H4'	H5'. H5"	H6/8	H2/5/Me
G11	5.99	2.756	2.822	4.952	4.332	3.879/ 3.987	8.058	-
C12	5.677	1.994	2.372	4.853	4.168	na/ na	7.333	5.378
G13	5.667	2.761	2.761	5.032	4.387	na/ na	7.892	-
A14	6.197	2.69	2.798	4.909	4.434	na/ na	8.064	7.737
**C15**	**5.772**	**2.176**	**2.504**	**na**	**4.164**	**4.17/ 4.298**	**7.191**	**5.025**
**|**								
**A16**	**5.587**	**2.594**	**2.701**	**4.963**	**na**	**na / na**	**7.783**	**6.84**
A17	6.085	2.583	2.793	5.003	4.43	na / na	8.073	7.602
C18	5.57	1.862	2.268	4.779	4.123	na/ 4.233	7.173	5.186
G19	5.881	2.58	2.682	4.969	4.332	na / na	7.86	-
C20	6.213	2.234	2.463	4.844	4.188	na / na	7.548	5.573

* The G6-C15 and T5-A16 base pairs. in bold. are flanking both faces of a nick.
na-not assigned.

Table S2. The ^{1}H NMR chemical shifts δ [ppm] of changes induced in free DNA decamer and **1** in D_2O (**1**:**3** ratio).

Base	H1'	H2'	H2"	H3'	H4'	H5'. H5"	H6/8	H2/5/Me
G1	-0.01	-0.001	- 0.006	-0.001	-0.011	0 / -0.024	-0.02	-
C2	0.00	-0.026	- 0.033	-0.007	-0.019	-	-0.014	-0.035
G3	-0.04	-0.029	- 0.048	-0.019	-0.016	-	-0.04	-
T4	0.00	-0.013	- 0.061	0.012	-0.041	-	0	-0.022
T5	**0.01**	**0.045**	**- 0.062**	**-0.005**	**-0.022**	**-**	**0.069**	**-0.020**
nick	-----------	-----------	-----------	-----------	-----------	-----------------------	-----------	-----------
G6	**-0.36**	**-0.326**	**- 0.081**	**0.02**	**-0.053**	**-0.08 / -0.26**	**0.009**	-
T7	-0.06	-0.029	- 0.051	-0.025	-0.115	-	-0.236	-0.027
C8	-0.04	-0.064	- 0.063	-0.03	-0.043	-	-0.048	-0.049
G9	-0.01	0.009	- 0.002	0.009	-0.001	-	-0.021	-
C10	-0.01	-0.022	0.002	-0.002	-0.013	-	0.012	-0.022

Base	H1'	H2'	H2"	H3'	H4'	H5'. H5"	H6/8	H2/5/Me
G11	-0.01	-0.017	-0.017	-0.007	-0.011	0 / -0.024	-0.02	-
C12	-0.01	-0.021	-0.013	0.003	-0.013	-	-0.034	-0.05
G13	0	0.023	-0.054	-0.015	-0.007	-	-0.028	-
A14	-0.02	0.009	-0.15	-0.122	-0.059	-	-0.094	**-0.06**
C15	**-0.09**	**0.238**	**0.142**	**-**	**0.02**	**-**	**0.035**	**-0.265**
A16	**0.057**	**0.020**	**-0.107**	**-0.045**	**-**	**-**	**-0.247**	**-0.218**
A17	-0.02	-0.056	-0.045	-0.034	-0.029	-	-0.088	**-0.101**
C18	-0.01	0.007	-0.011	-0.026	-0.019	-	0.006	-0.014
G19	0.012	-0.034	-0.01	-0.021	-0.022	-	-0.008	-
C20	0	-0.022	0.002	-0.002	-0.013	-	-0.007	-0.017

Table S3. The ^{1}H NMR chemical shift changes in SN38 derivative **1** after addition to DNA.

**Compound 1**	**Free; δ [ppm]**	**Complex 1+2; δ [ppm]**	**Δ [ppm]**	**Δ [Hz]**
19-CH_3	0.972	0.98	0.008	4
23-CH_3	1.534	1.422	-0.112	-56
18-CH_2	1.954	1.908	-0.046	-23
22-CH_2	3.113	2.986	-0.127	-63.5
25-NCH_2	3.358	3.404	0.046	23
26-OCH_2	3.958	3.975	0.017	8.5
24-CH_2	4.624	4.615	-0.009	-4.5
24-CH_2	4.707	4.615	-0.092	-46
5-CH_2	4.991	no	no	no
17-CH_2	5.358	5.456	0.098	49
17-CH_2	5.5	5.456	-0.044	-22
14-CH	7.146	7.038	-0.108	-54
11-CH	7.303	7.136	-0.167	-83.5
12-CH	7.683	7.408	-0.275	-137.5

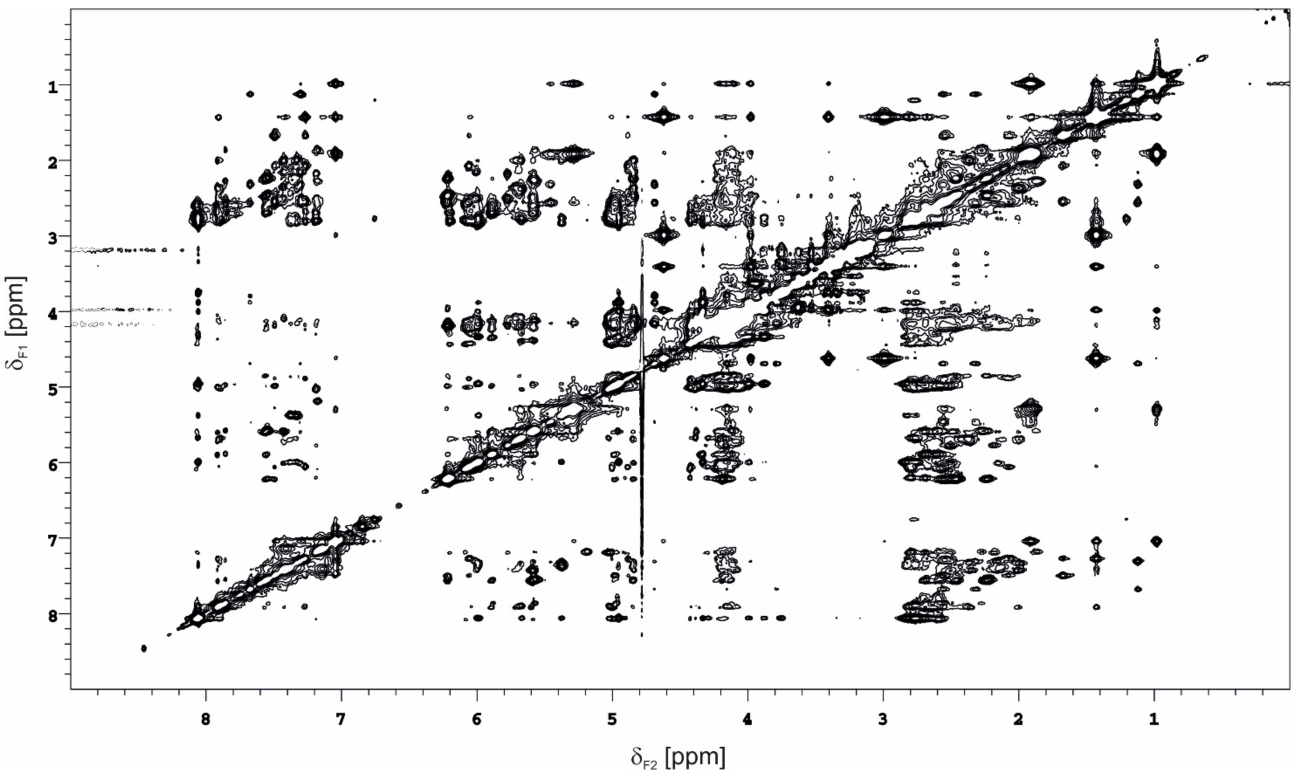

Figure S3 The NOESY spectrum of the sample 1+2B (**1**:**3** ratio).

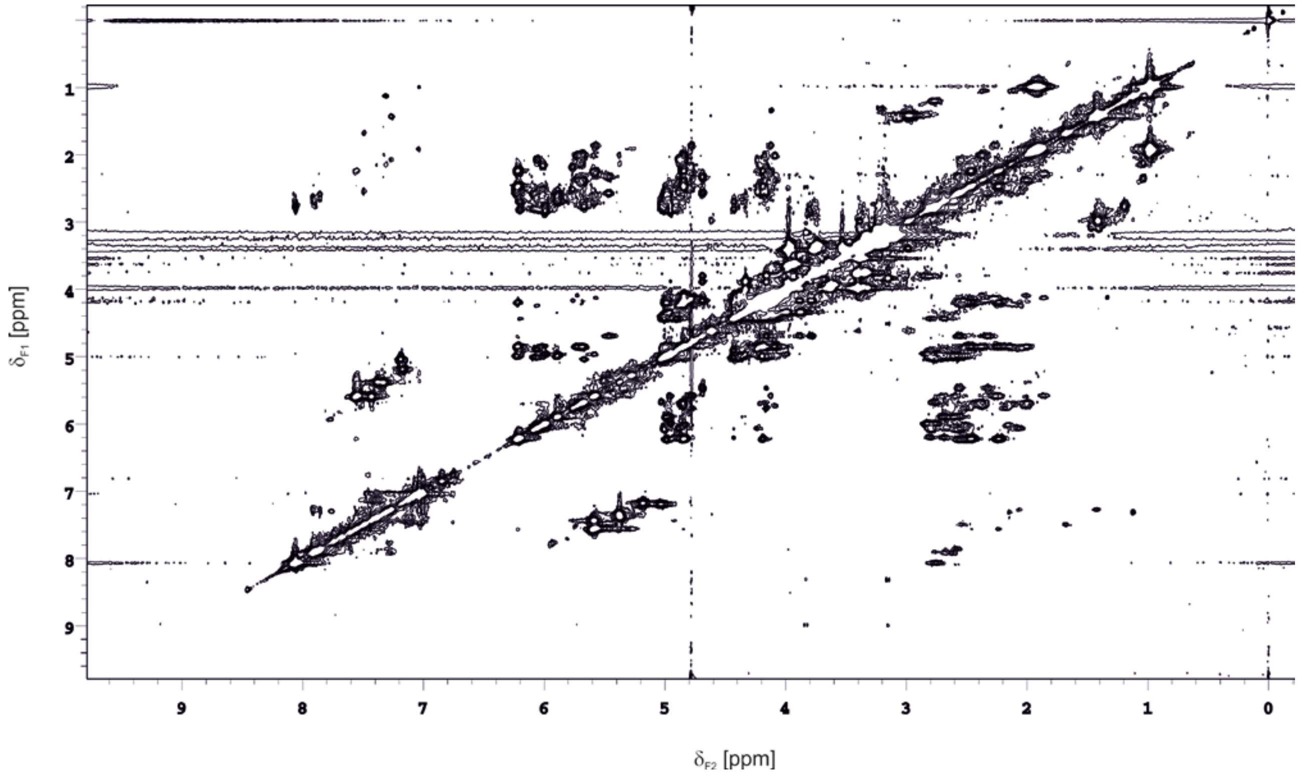

Figure S4 The TOCSY spectrum of the sample 1+2B (**1**:**3** ratio).

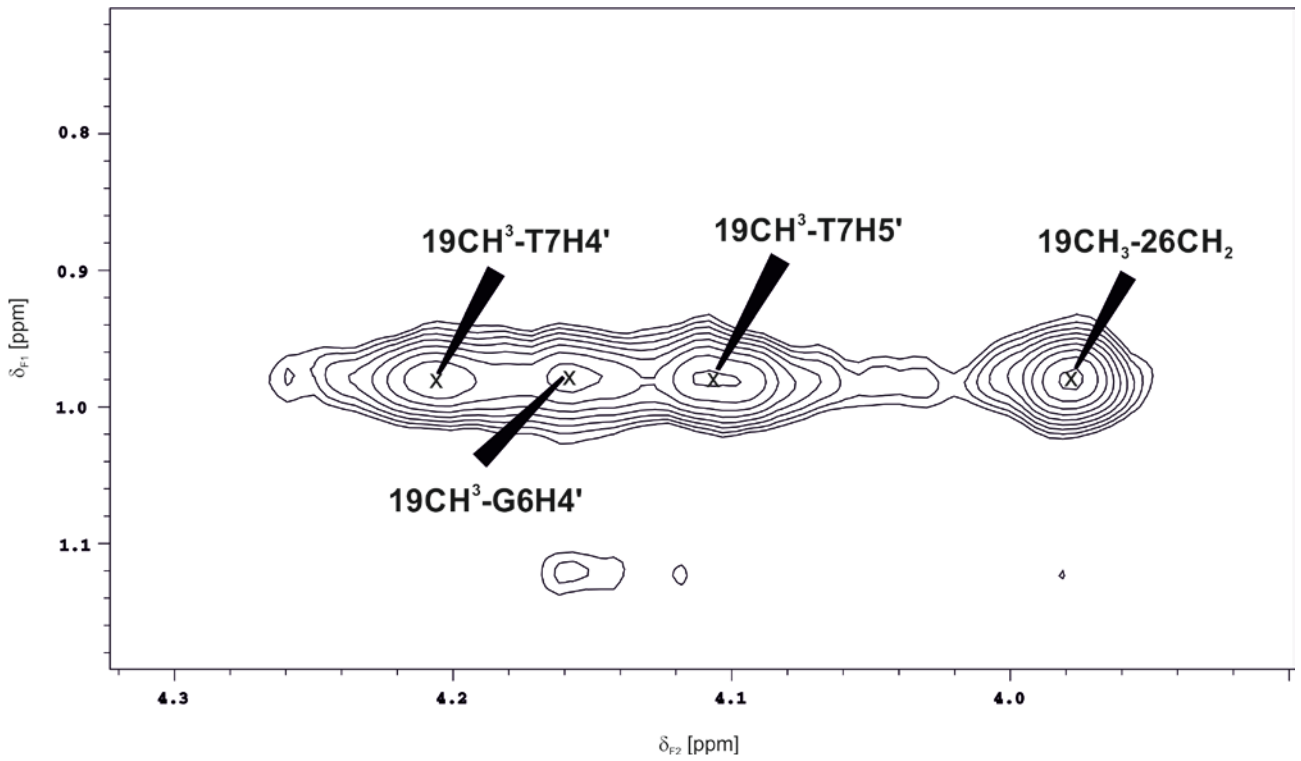

Figure S5 The example of intermolecular cross-peaks in complex 1+2B before filtering the reaction solution.

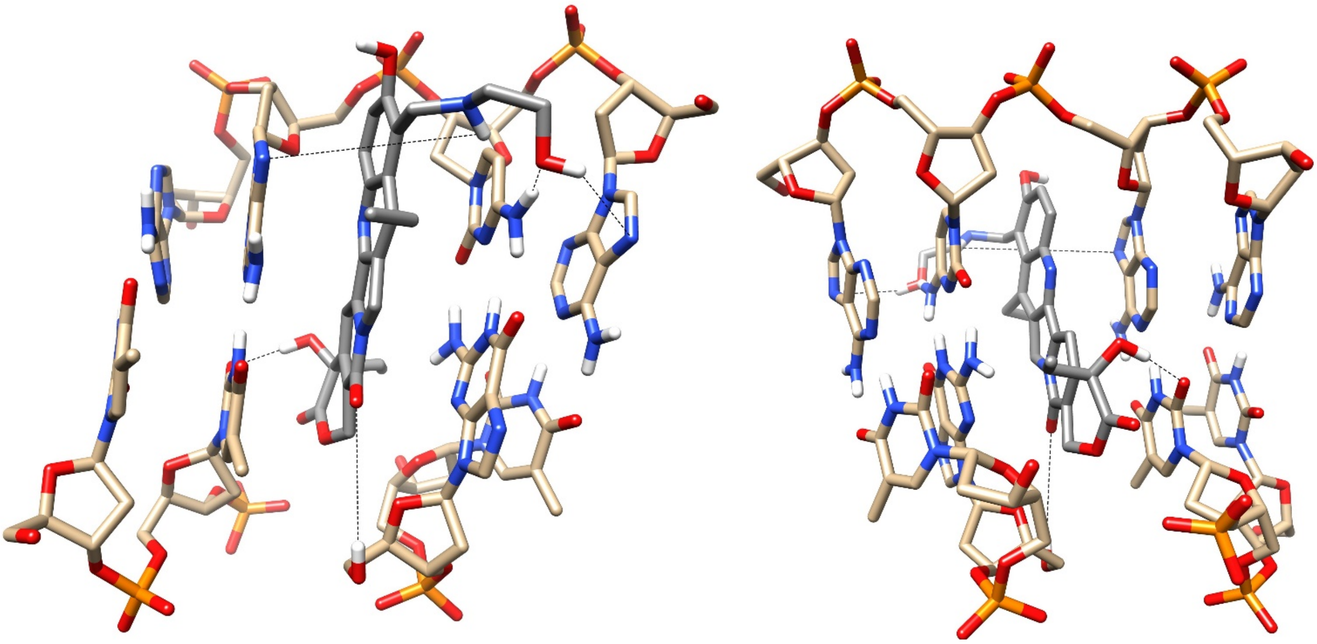

Structure 1

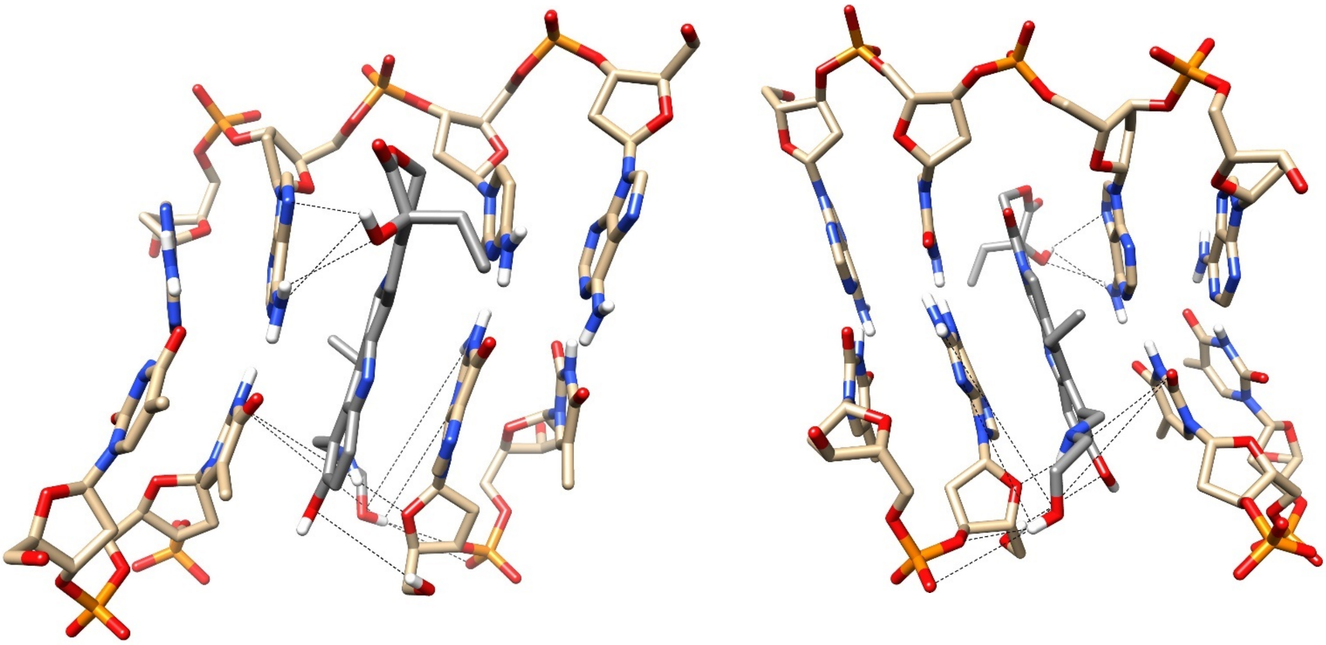

Structure 2

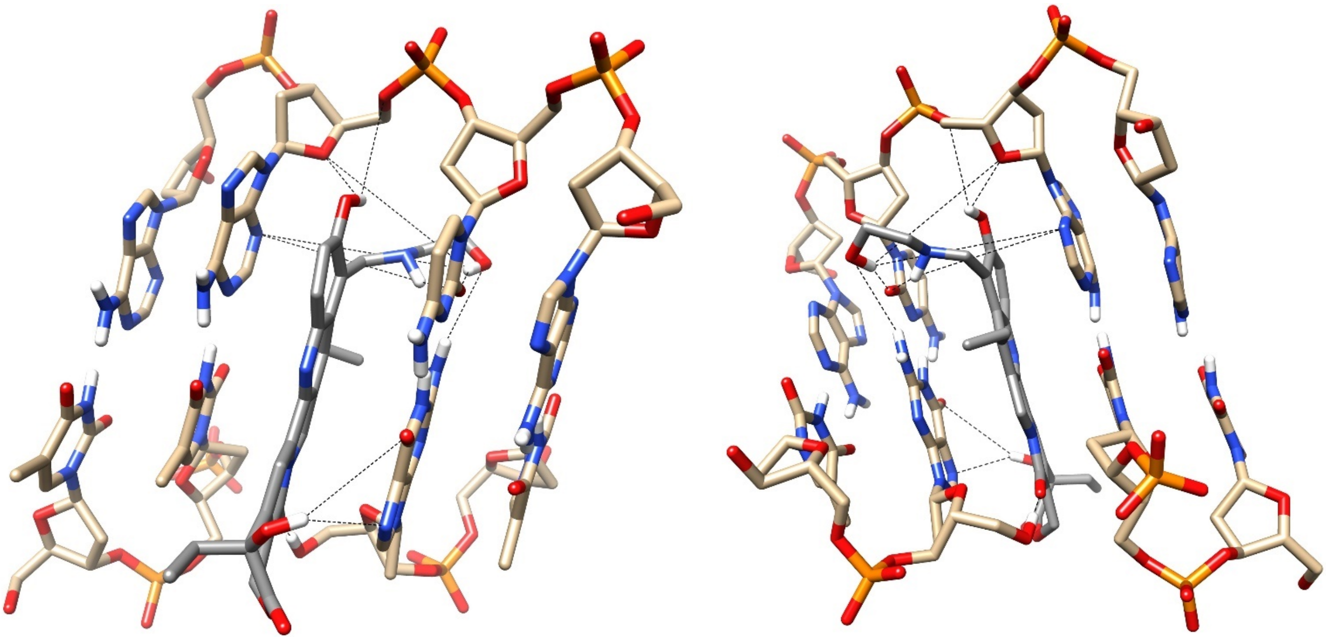

Structure 3

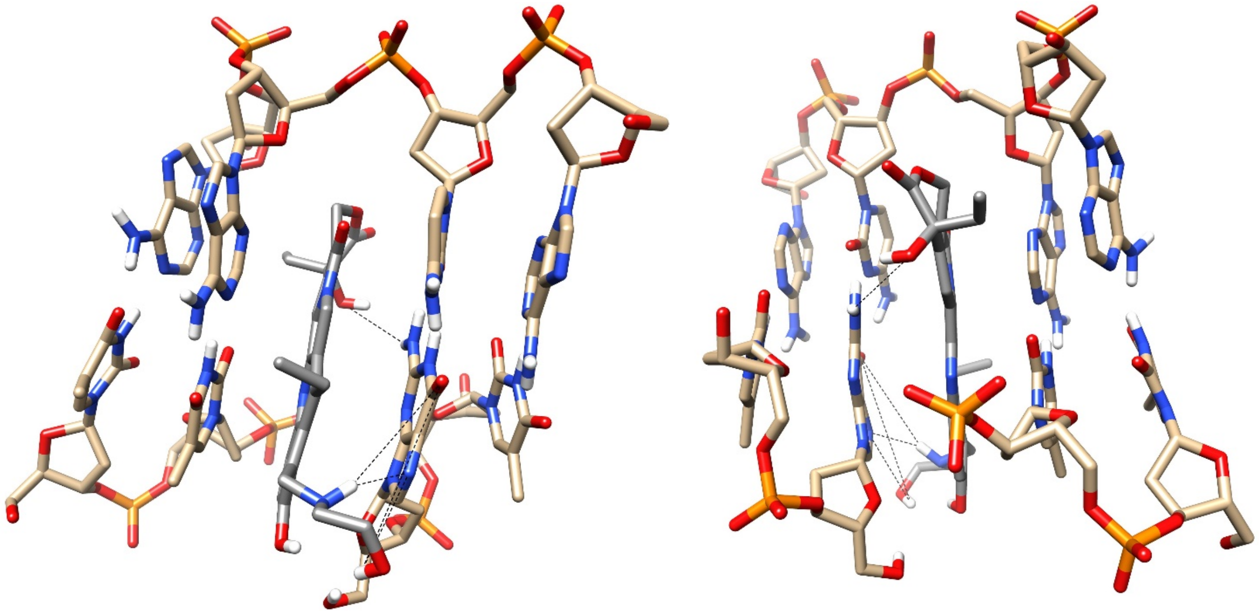

Structure 4

Figure S6 The hydrogen bonding in structures best representing the most populated cluster obtained by cluster analysis in a molecular complex 1+2A.

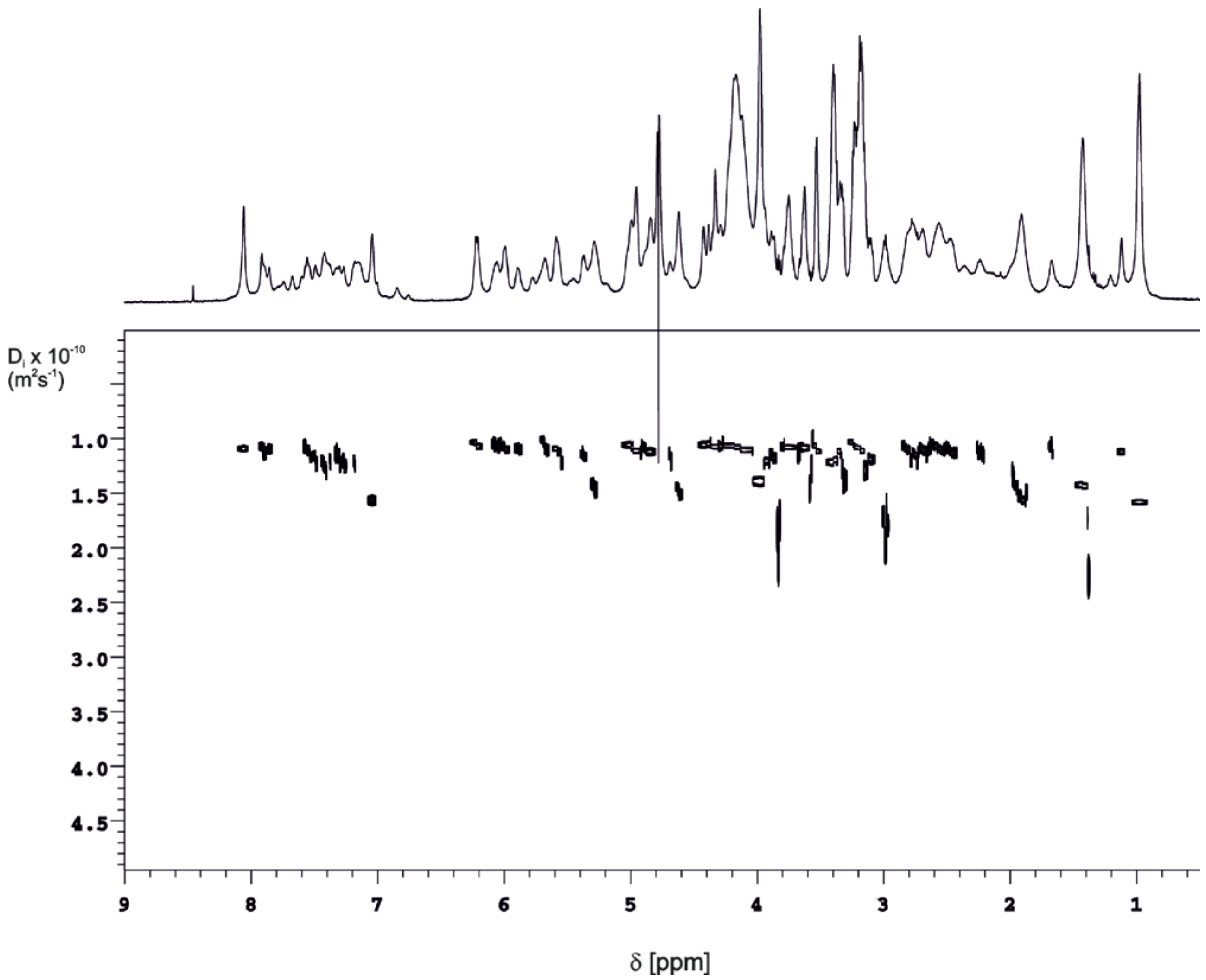

Figure S7 The DOSY spectrum presenting mixture of **1** with **2B** at the start of the reaction in buffered D_2O. 25 mM NaCl /25 mM K_3PO_4. at pH 6.

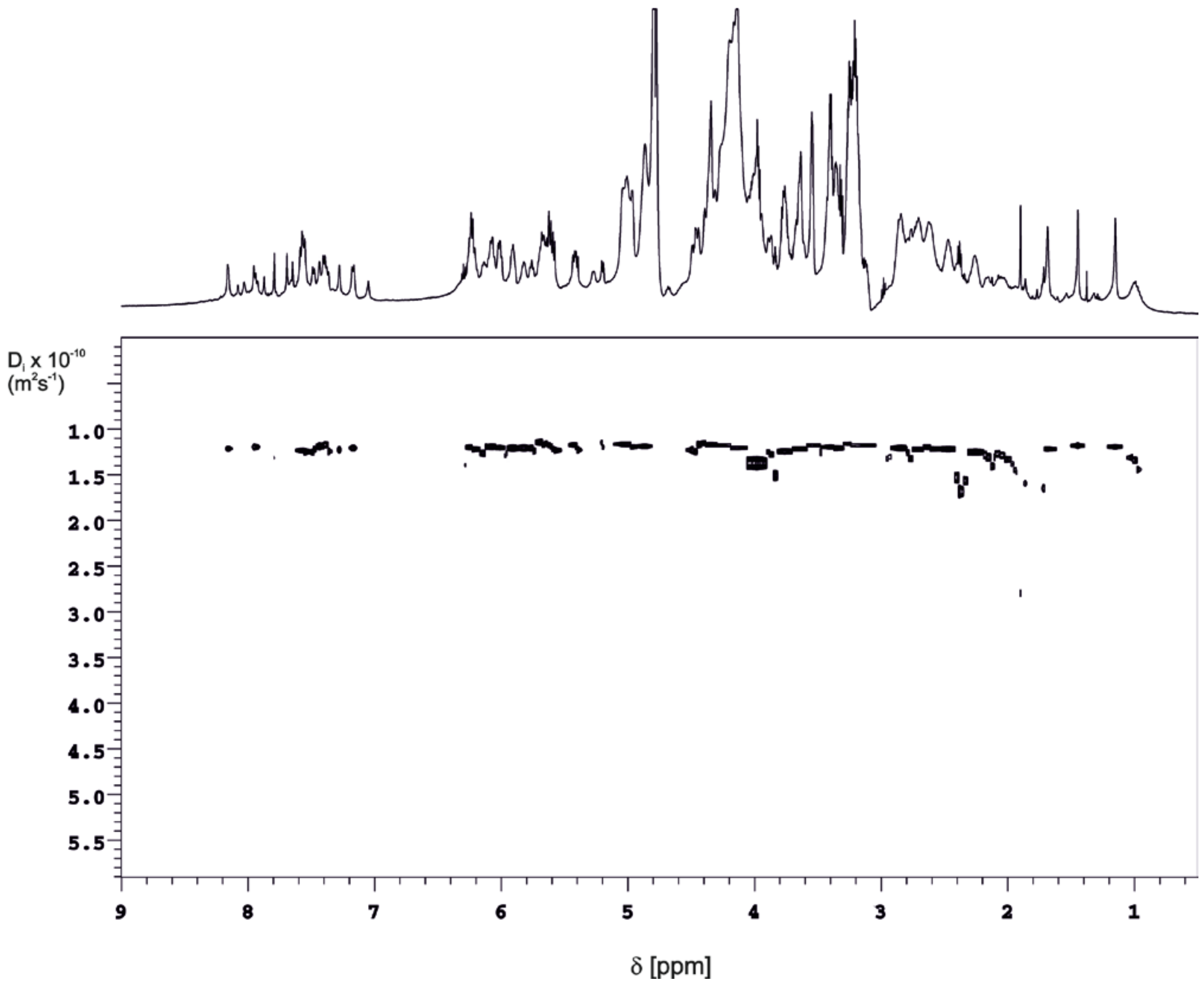

Figure S8. The full DOSY spectrum of a 1:1 complex of **1** and **2B** in D_2O buffer. pH 6. 25°C.

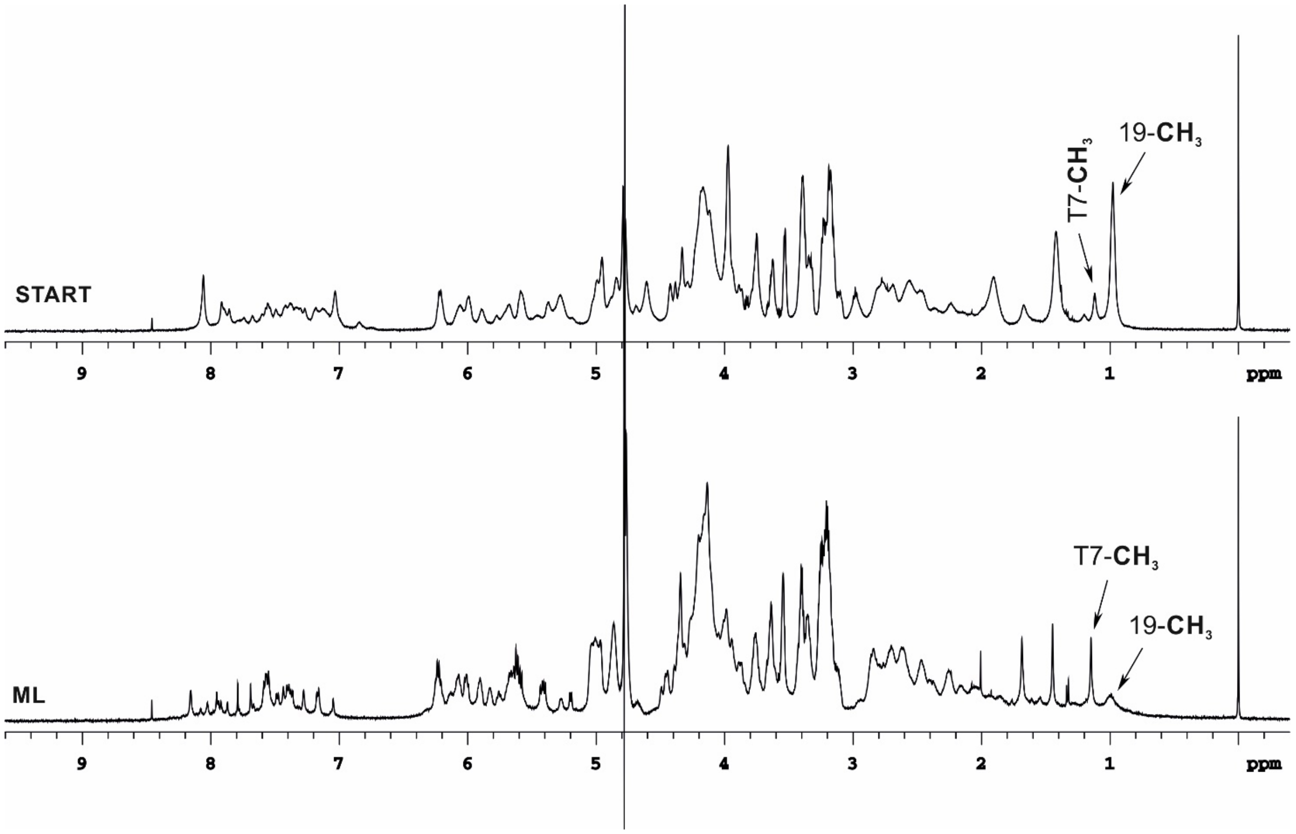

Figure S9 The shape of 1D NMR spectrum of reaction mixture of **2B** with **1** at the start and in a mother liquor ML after filtering of reaction solution.

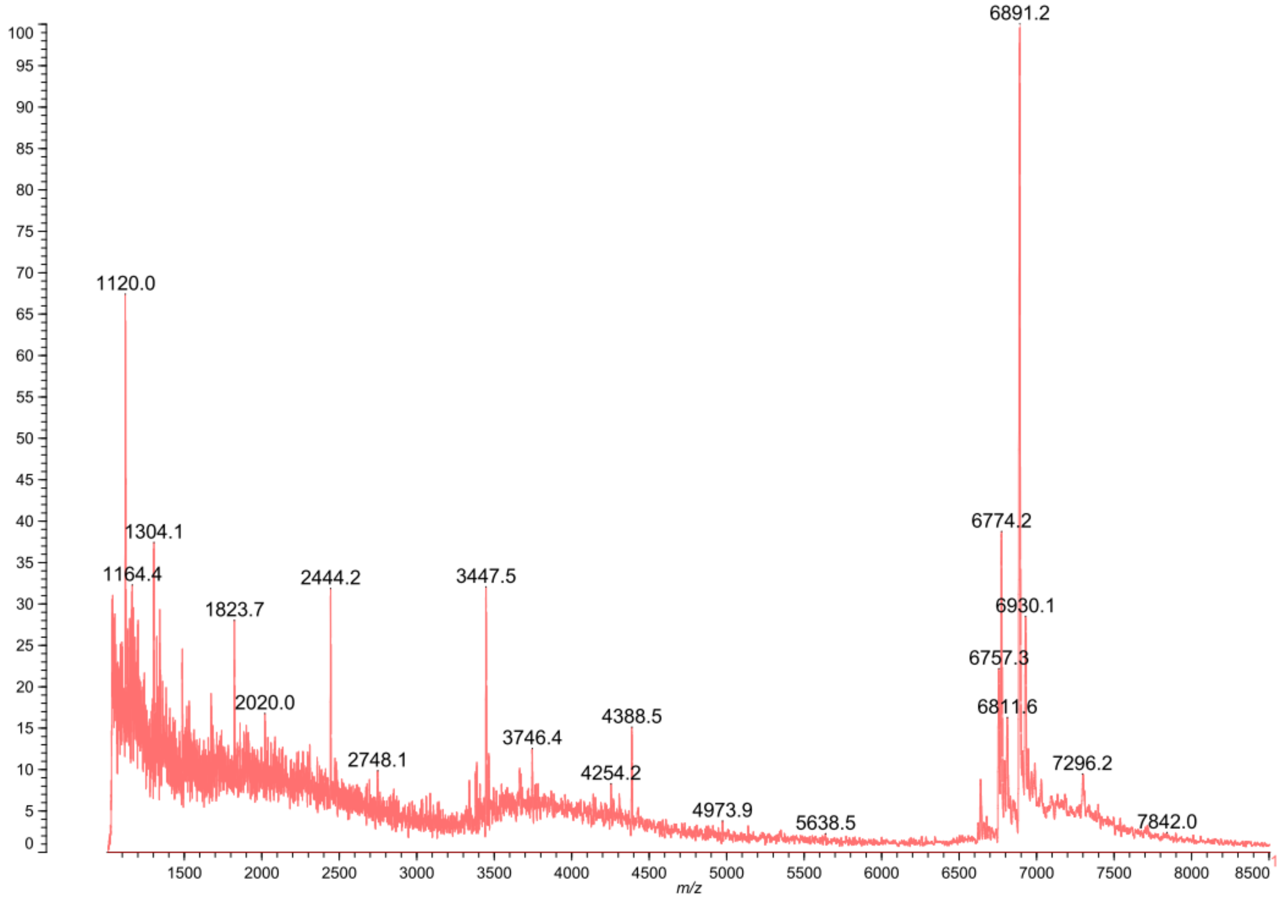

Figure S10 The MALDI-ToF mass spectrum of lyophilized ML showing neat DNA decamer (2B) (*m/z*=6891.2) and alkylated biohybrid with compound **1** (*m/z*=7296.2).
